# Peripheral vascular catheter use in Latin America (the vascular study): A multinational cross-sectional study

**DOI:** 10.3389/fmed.2022.1039232

**Published:** 2023-01-04

**Authors:** Rachel M. Walker, Maria Paula Oliveira Pires, Gillian Ray-Barruel, Marie Cooke, Gabor Mihala, Silvia Schoenau Azevedo, Maria Angelica Sorgini Peterlini, Marcelle Di Angelis Ambar Felipe, Cirlia Petrona Álvarez, Marcela Quintanilla, Martha Claudia Corzo, Gabriela Cortez Villareal, Eliazib Nataren Cigarroa, Mavilde L. G. Pedreira, Claire M. Rickard

**Affiliations:** ^1^School of Nursing and Midwifery, Griffith University, Brisbane, QLD, Australia; ^2^Alliance for Vascular Access Teaching and Research (AVATAR), School of Pharmacy and Medical Sciences and School of Nursing and Midwifery, Griffith University, Brisbane, QLD, Australia; ^3^Division of Surgery, Princess Alexandra Hospital, Brisbane, QLD, Australia; ^4^Pediatric Nursing Department, Escola Paulista de Enfermagem, Universidade Federal de São Paulo, São Paulo, Brazil; ^5^School of Nursing, Midwifery and Social Work, The University of Queensland, Brisbane, QLD, Australia; ^6^Metro North Hospitals and Health Service, Herston Infectious Diseases Institute (HeIDI), Brisbane, QLD, Australia; ^7^Centre for Applied Health Economics, Menzies Health Institute Queensland, Griffith University, Brisbane, QLD, Australia; ^8^Infection Control Service, Asociación de Terapia de Infusión y Seguridad del Paciente (ATISPA), Buenos Aires, Argentina; ^9^Sociedad Chilena de Terapia de Intravenosa (SOCHITEIN), Santiago, Chile; ^10^Asociación Colombiana de Terapia Intravascular (ACOTEIN), Bogotá, Colombia; ^11^National Institute of Cardiology Ignacio Chávez, Directorate of Quality and Health Education of the Ministry of Health of Mexico, Ciudad de México, Mexico; ^12^Dr. Jesús Gilberto Gómez Maza Hospital, Health Services of the State of Chiapas, Tuxtla Gutiérrez, Mexico; ^13^National Council for Scientific and Technological Development (CNPq), Brasília, Brazil

**Keywords:** vascular access device, vascular catheter, intravenous infusion, cross-sectional study, prevalence study, multinational perspectives

## Abstract

**Background:**

Peripheral intravenous catheter (PIVC) insertion is one of the most common clinical procedures worldwide, yet little data are available from Latin America. Our aim was to describe processes and practices regarding PIVC use in hospitalized patients related to hospital guidelines, characteristics of PIVC inserters, prevalence of PIVC complications, and idle PIVCs.

**Methods:**

In 2019 we conducted a multinational, cross-sectional study of adult and pediatric patients with a PIVC in hospitals from five Latin American countries: Argentina, Brazil, Chile, Colombia, and Mexico. We used two data collection tools to collect hospital guidelines and patient-specific data on the day of the study. The vessel health and preservation (VHP) model guided synthesis of the study aims/questions and suggested opportunities for improvement.

**Results:**

A total of 9,620 PIVCs in adult (86%) and pediatric inpatients in 132 hospitals were assessed. Routine replacement 8–72 hourly was recommended for adults in 22% of hospitals, rather than evidence-based clinical assessment-based durations, and 69% of hospitals allowed the use of non-sterile tape rather than the international standard of a sterile dressing. The majority (52%) of PIVCs were inserted by registered nurses (RNs), followed by nursing assistants/technicians (41%). Eight percent of PIVCs had pain, hyperemia, or edema, 6% had blood in the extension tubing/connector, and 3% had dried blood around the device. Most PIVCs had been inserted for intravenous medications (81%) or fluids (59%) in the previous 24 h, but 9% were redundant.

**Conclusion:**

Given the variation in policies, processes and practices across countries and participating hospitals, clinical guidelines should be available in languages other than English to support clinician skills and knowledge to improve PIVC safety and quality. Existing and successful vascular access societies should be encouraged to expand their reach and encourage other countries to join in multinational communities of practice.

## 1. Introduction

Peripheral intravenous catheter (PIVC) insertion is one of the most common clinical procedures worldwide (1, 2). Approximately 70% of all hospitalized patients in developed world health settings require PIVC access for a broad range of clinical applications vital for patient diagnosis and treatment (1, 2).

Usually inserted in upper extremity veins (3), PIVCs are frequently associated with complications during insertion and ongoing management, leading to delays in diagnosis and treatment (3, 4), and compromising patient experience (5–7). Quality indicators include partial displacement, accidental removal, phlebitis, occlusion, infiltration, blood leakage around the device insertion site, and local or bloodstream infection (8, 9). Adverse events related to PIVCs have significant ramifications for patient safety and quality of care (9, 10).

Healthcare systems differ between Latin American countries, influenced by diverse political and economic/social contexts and agendas (9). Reduced funding for research and competing research priorities also create significant barriers to knowledge and practice related to vascular access (10), as does the shortage of healthcare professionals with training in clinical research.

Information about PIVC use in patients admitted to Latin American hospitals is scarce and has limited focus, such as infection only (11) or a predominance of top-tier facilities in major population centers (1), which may not be reflective of the large diversity within these nations. Despite individual studies/hospital reports indicating PIVC complications are common, limited data examines why these complications occur.

Although the evidence on vascular access devices is rapidly expanding, as evidenced by international studies (1, 11, 12), much of the data comes from high income countries whose health services are supported by significant allocation of their total national expenditure. The World Health Organization cites the limited allocation of funding to healthcare in low and middle-income regions, including Latin America, as a contributing factor to the high mortality rate related to nosocomial infections, estimated to be 20 times higher than in high income countries (13).

While there are growing calls to promote health research *via* integrated approaches to funding and resources for health research (14), there is a paucity of Latin American data on PIVC use. The aim of the VAScular Catheter Use in Latin AmeRica (VASCULAR) study was to describe processes and practices regarding PIVC use in hospitalized patients in five Latin American countries and to identify opportunities for change and improvement based on the following research questions:

1. What are the hospital policies and processes for PIVC insertion and maintenance?
2. What are the PIVC inserter characteristics (level of education, training)?
3. What is the prevalence of PIVC complications (pain, redness/hyperemia, and edema)?
4. What is the prevalence of redundant/idle PIVCs (calculated by number of PIVCs not used for intravenous therapy in a 24-h period)?

## 2. Material and methods

### Ethics and agreements

The study was initially approved by the Griffith University Human Research Ethics Committee (Ref No: 2018/292). Hospitals in each country were then invited to participate by our Associate Investigators, recognized as expert clinicians in vascular access in their home countries (refer to the stratified sampling frame in Supplementary File 1). Hospitals interested in participating were provided with the original ethics approval from Griffith University and additional information about the study including data management and confidentiality. Hospitals were required to obtain ethics approvals according to their hospital, regional or national requirements, and all participants or their guardians provided informed written consent. Data from participating hospitals were not shared and remained the property of the hospital. Deidentified results were therefore reported by country, not by hospital. However, while all data collected remains the property of the participating hospitals, respectively, they agreed to follow the study protocol and authorship agreement to ensure results of the main study were written and published in a peer-reviewed journal prior to local publication and/or dissemination. Each participating hospital was acknowledged as a contributor to the study. No financial aid was provided to the hospitals (Supplementary Files 2,3).

### Study design and setting

A stratified sampling frame was developed using population data per region (Supplementary File 1). This broad approach enabled a variety of hospital types in both metropolitan and regional areas to be involved in the study. We recruited hospitals from five countries (Argentina, Brazil, Chile, Colombia, and Mexico) to perform an audit of all PIVCs in place on one calendar day. The participating countries are economic leaders in the region with large urban populations and the highest gross domestic productivity in Latin America (15, 16). Hospitals were invited to participate *via* a network of professional in-country leaders, key contacts, vascular access organizations, newsletters, trade publications, conference presentations, social media, and word-of-mouth. As collaborations are an essential component for the construction and maintenance of health research capacity in Latin America (17), popular online social media platforms such as Facebook and Instagram were used to promote the study.

Preparation for the study commenced late in 2017. Each country had their local recruiting coordinator (Associate Investigator: AI). Significant time was taken to develop relationships with in-country AIs who participated directly with lead investigators *via* fortnightly Skype (Skype Technologies) team meetings. To overcome language barriers, multilingual research nurse assistants were employed in Brazil to translate during meetings and coordinate preparation of study documents, data collection tools including an electronic database (REDCap: Research Electronic Data Capture, developed at Vanderbilt University and hosted by Universidade Federal de São Paulo) (18, 19), and institutional ethics approvals.

Initially, a screening tool was used to guide and record recruitment of hospitals in each country. In-country AIs with significant experience in vascular access recruited hospitals from December 2018 to September 2019, and provided advice to hospitals on project aims, ethics, and data collection processes. This information (hospital name, key contact person, ethics, and authorship approvals) was returned to research assistants in Brazil who entered this data into a master database.

### Data sources/Measurement

Two data collection forms were developed to ensure assessment/selection, insertion, maintenance, and evaluation variables associated with the PIVC continuum were captured (20). The data collection forms were initially developed in English then translated into Portuguese and Spanish. While not formally validated, variable selection was informed by a previously conducted international study (1) and transcultural translation was rigorously undertaken within the multinational team to ensure local terminology was used appropriately for optimal understanding in each country.

Audit data were collected from January to November 2019. The choice of date for the audit was decided by individual hospitals in consultation with the in-country AI, subject to staffing, workload, and other local circumstances. The sampling approach was determined by the capacity of each hospital and the profile/number of patients with a PIVC. Hospitals were encouraged to audit as many PIVCs as possible on the day of data collection, ideally the entire hospital.

Once a participating hospital had been recruited, a site information survey was completed by the local key contact or a clinical leader in nursing or medicine. This form captured general data such as the hospital location (region, state, or province) and specific organizational data such as: number of beds, the existence and use of PIVC insertion/maintenance guidelines within the organization, clinicians responsible for inserting PIVCs, use of PIVC insertion packs, type of PIVCs used, cleaning solutions and dressings routinely used, insertion techniques, use of tools for assessing phlebitis and other complications, staff training, and frequency of routine PIVC audits. Once completed, site information survey data were entered directly into REDCap by the person completing the form or returned electronically to be entered into the database by research assistants in Brazil (Supplementary File 4).

### Participants

All acute hospitalized adult and pediatric patients with a PIVC on the day of the audit were eligible for recruitment. Sample size was not pre-determined, due to variable availability of participants. Following a written informed consent process with eligible adult and pediatric patients (or their proxy), the data collection form gathered information *via* a visual inspection of the PIVC and referral to patient documentation.

### Variables

Demographic data collected included gender, date of birth, location, and health specialty. While there was a focus on key aims such as identifying device redundancy and PIVC complications, data about the insertion and management of the PIVC were also collected. This included, for example, identifying the clinician category who inserted the device, number of insertion attempts, anatomical site of insertion, type/gauge of PIVC, as well as dressings, securements, connectors, and tubing used (Supplementary File 5). Reasons for non-participation were not documented.

Data were collected in two ways: directly online *via* REDCap, or on paper with data entered into the electronic database by health professionals who oversaw the data collection at each site. Throughout the data collection period, data were checked for accuracy and quality by a central team based in Brazil. In some cases, colleagues at specific data collection sites were contacted for clarification.

### Guiding framework

The vessel health and preservation (VHP) model guided synthesis of the study aim and research questions (20, 21). Both a framework and clinical pathway developed *via* an integration of guidelines and functions, VHP provided a structured process to enable the systematic analysis of the study results with the goal of increasing quality indicators and reducing PIVC complications (20, 21). With education at its core, the model identifies key practice quadrants for the life of vascular access devices, as well as evidence-based recommendations to reduce the risk of PIVC complications to ensure patient safety.

**FIGURE 1:**
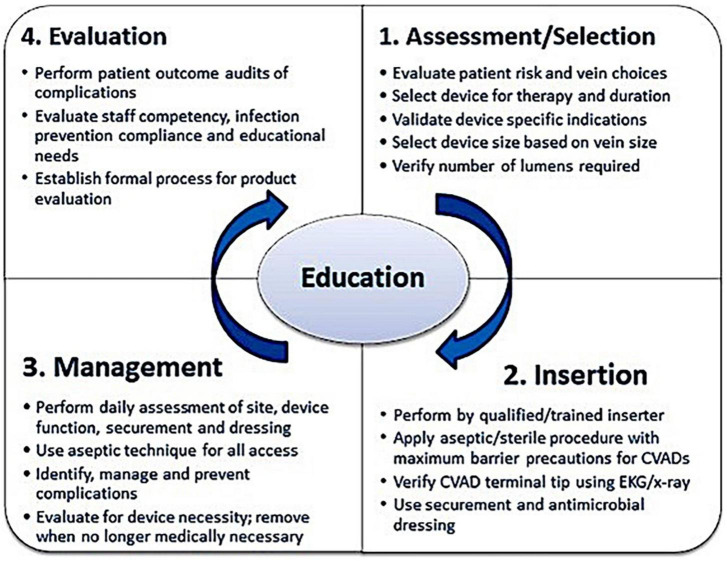
Vessel health and preservation (VHP) model (21). Used with permission.

As outlined in Figure 1, the VHP quadrants identify the functions required during the PIVC continuum (21). When used in combination, quadrant functions may contribute to a wider program of PIVC quality and patient safety. However, for this study we inverted the model to reflect order of data collection and research questions. As such, we commenced with Quadrant 4 to acknowledge the initial survey of organizational practice and guideline recommendations completed by participating hospitals followed by characteristics of PIVC use identified in the audit of patients in clinical settings (Quadrants 1–3).

Quadrant 4, *Evaluation*, adopts a global approach to PIVC care: one that acknowledges the need to review current vascular access practices within the organization and identify practice deficits and complications to determine targeted training requirements based on the latest evidence. This can be achieved *via* a culture of regular audit, surveillance, and patient satisfaction measures, as well as tailored education for clinicians responsible for PIVC insertion.

The goal of Quadrant 1, *Assessment/selection*, is to ensure the right PIVC is inserted into the right patient, at the right time, determined by patient diagnosis and treatment plan. As such, the treating clinician or team should assess the patient’s condition and vasculature, select the most appropriate vein and PIVC size.

Quadrant 2, *Insertion*, should be performed by a trained clinician who can identify the best vein for placement to reduce the number of insertion attempts. The inserted PIVC should be secured with an appropriate dressing. Education for inserters should include infection prevention practices with ongoing and regular insertion training under the direct supervision of a vascular access expert.

Quadrant 3 outlines the *Assessment and management* to maintain PIVC function, as well as securement of the device and dressing. Functions include visual assessment to identify complications, flushing the line to ensure patency, and replacing the dressing if loose or soiled. Other management considerations include use of aseptic non-touch technique when accessing the PIVC and reassessment of the need for the device. These data were mapped to quadrants based on the study research questions (Supplementary File 6) (21).

### Statistical analysis

Data were downloaded from REDCap to Stata 15 (StataCorp, College Station, TX, USA), hosted on Griffith University computers, for data management and analysis. Sharing of site-specific research data with the research teams was conducted using the ownCloud software (90411 Nürnberg, Germany), hosted by Griffith University. Results were analyzed descriptively by hospital population, redundant PIVCs, and complications. Descriptive comparisons were also made between countries. Frequencies and percentages were calculated. Data forms that were 95% incomplete were considered input errors and excluded from analysis. Missing data were not imputed. The STROBE (Strengthening the Reporting of Observational Studies in Epidemiology Statement) guidelines for cross-sectional studies were followed (22, 23), and results are presented according to these recommendations (Supplementary File 7).

## 3. Results

This study included a total of 9,620 PIVCs in 8,059/9,330 (86%) adult and 1,271/9,330 (14%) pediatric patients in 132 hospitals across five countries. Results are reported according to our organization of the quadrants of the VHP model, firstly reporting hospital level data, followed by patient level data according to study questions.

### Hospital policies and processes for PIVC insertion and maintenance

From the hospital level data, registered nurses (RNs) were the largest group of PIVC inserters across all 132 sites (130 sites, 98%), following by nursing technicians (69 sites, 52%) and nursing assistants (49 sites, 37%). The most common PIVC insertion technique in hospital guidelines was the aseptic non-touch technique (91 sites, 69%). However, the 31 Mexican hospitals had a higher frequency of strict asepsis where sterile gloves were used (15 sites, 48%). Approved solutions to clean the skin prior to PIVC insertion included 0.9% sodium chloride (130 sites, 98%), povidone-iodine (129 sites, 98%), and chlorhexidine 1% in 70% alcohol (128 sites, 97%). Participating hospitals also offered a variety of dressing options for PIVC sites, including chlorhexidine-impregnated dressings (125 sites, 95%), non-sterile tape (121 sites, 92%), and sterile gauze and tape (107 sites, 81%) (Table 1).

**TABLE 1:** Characteristics of current practice and guideline recommendations (site information survey).

	*N*	Argentina	Brazil	Chile	Colombia	Mexico	Total
Number of hospitals	132	23 (17%)	46 (35%)	18 (14%)	14 (11%)	31 (23%)	132 (100%)
**Frequency of PIVC replacement in adults**	**115**	***n* = 16**	***n* = 37**	***n* = 18**	***n* = 14**	***n* = 30**	
8–72 h		2 (12%)	5 (14%)	4 (22%)	2 (14%)	12 (40%)	25 (22%)
72–96 h		4 (25%)	26 (70%)	10 (56%)	6 (43%)	9 (30%)	55 (48%)
Clinically indicated		9 (56%)	2 (5%)	2 (11%)	5 (36%)	3 (10%)	21 (18%)
Other		1 (6%)	4 (11%)	2 (11%)	1 (7%)	6 (20%)	14 (12%)
**Frequency of PIVC replacement in children**	**115**	***n* = 16**	***n* = 37**	***n* = 18**	***n* = 14**	***n* = 30**	
8–72 h		0 (0%)	3 (8%)	0 (0%)	0 (0%)	3 (10%)	6 (5%)
72–96 h		1 (6%)	9 (24%)	0 (0%)	2 (14%)	8 (27%)	20 (17%)
Clinically indicated		11 (69%)	19 (51%)	16 (89%)	10 (71%)	12 (40%)	68 (59%)
Other		4 (25%)	6 (16%)	2 (11%)	2 (14%)	7 (23%)	21 (18%)
**Recommended frequency of assessment** ^a,b^		***n* = 13**	***n* = 25**	***n* = 16**	***n* = 12**	***n* = 23**	
Every 4 h or more often	89	0 (0%)	3 (12%)	3 (19%)	0 (0%)	3 (13%)	9 (10%)
Every 6 h	89	4 (31%)	7 (28%)	0 (0%)	4 (33%)	2 (9%)	17 (19%)
Every 8 h	89	4 (31%)	4 (16%)	0 (0%)	1 (8%)	16 (70%)	25 (28%)
Every 12 h	89	0 (0%)	0 (0%)	9 (56%)	4 (33%)	1 (4%)	14 (16%)
Every 24 h	89	0 (0%)	5 (20%)	6 (38%)	1 (8%)	1 (4%)	13 (15%)
Whenever the catheter is used	89	7 (54%)	8 (32%)	1 (6%)	2 (17%)	5 (22%)	23 (26%)
Other	89	0 (0%)	3 (12%)	1 (6%)	1 (8%)	1 (4%)	6 (7%)
**Recommended PIVC insertion technique**	**132**	***n* = 23**	***n* = 46**	***n* = 18**	***n* = 14**	***n* = 31**	
Aseptic (non-touch technique)		18 (78%)	39 (85%)	13 (72%)	8 (57%)	13 (42%)	91 (69%)
Strictly aseptic		3 (13%)	0 (0%)	4 (22%)	1 (7%)	15 (48%)	23 (17%)
Clean		2 (9%)	7 (15%)	1 (6%)	5 (36%)	3 (10%)	18 (14%)
**PIVC inserters** ^a,b,c^		***n* = 23**	***n* = 46**	***n* = 18**	***n* = 14**	***n* = 31**	
Registered nurse	132	23 (100%)	44 (96%)	18 (100%)	14 (100%)	31 (100%)	130 (98%)
Nursing technician	132	10 (43%)	46 (100%)	3 (17%)	2 (14%)	8 (26%)	69 (52%)
Nursing assistant	132	6 (26%)	25 (54%)	0 (0%)	14 (100%)	4 (13%)	49 (37%)
Nursing student	132	2 (9%)	24 (52%)	5 (28%)	6 (43%)	8 (26%)	45 (34%)
Doctor/resident	132	6 (26%)	24 (52%)	5 (28%)	2 (14%)	7 (23%)	44 (33%)
Specialist intravenous team	132	1 (4%)	7 (15%)	0 (0%)	2 (14%)	12 (39%)	22 (17%)
Medical student	132	1 (4%)	15 (33%)	0 (0%)	0 (0%)	0 (0%)	16 (12%)
Other	132	2 (9%)	0 (0%)	6 (33%)	4 (29%)	2 (6%)	14 (11%)
**Cleaning solution used for insertion** ^a,b^		***n* = 23**	***n* = 46**	***n* = 18**	***n* = 14**	***n* = 31**	
0.9% sodium chloride	132	22 (96%)	46 (100%)	18 (100%)	14 (100%)	30 (97%)	130 (98%)
Povidone-iodine in alcohol	132	22 (96%)	46 (100%)	18 (100%)	14 (100%)	29 (94%)	129 (98%)
Chlorhexidine 1% in 70% alcohol	132	22 (96%)	44 (96%)	18 (100%)	13 (93%)	31 (100%)	128 (97%)
Chlorhexidine without alcohol	132	21 (91%)	43 (93%)	15 (83%)	14 (100%)	29 (94%)	122 (92%)
Povidone-iodine without alcohol	132	19 (83%)	46 (100%)	18 (100%)	14 (100%)	20 (65%)	117 (89%)
Chlorhexidine 0.5% in 70% alcohol	132	21 (91%)	33 (72%)	16 (89%)	14 (100%)	30 (97%)	114 (86%)
Chlorhexidine 2% in 70% alcohol	132	11 (48%)	33 (72%)	16 (89%)	4 (29%)	14 (45%)	78 (59%)
70–75% alcohol	132	7 (30%)	6 (13%)	0 (0%)	9 (64%)	6 (19%)	28 (21%)
Other	132	22 (96%)	45 (98%)	18 (100%)	14 (100%)	31 (100%)	130 (98%)
**Dressing type** ^a,b^		***n* = 23**	***n* = 46**	***n* = 18**	***n* = 14**	***n* = 31**	
Chlorhexidine-impregnated	132	20 (87%)	46 (100%)	16 (89%)	14 (100%)	29 (94%)	125 (95%)
Sterile tape	132	23 (100%)	37 (80%)	18 (100%)	14 (100%)	29 (94%)	121 (92%)
Sterile gauze and tape	132	13 (57%)	39 (85%)	16 (89%)	13 (93%)	26 (84%)	107 (81%)
Non-sterile tape	132	16 (70%)	24 (52%)	16 (89%)	8 (57%)	27 (87%)	91 (69%)
Window transparent polyurethane	132	16 (70%)	36 (78%)	3 (17%)	6 (43%)	14 (45%)	75 (57%)
Borderless transparent polyurethane	132	6 (26%)	21 (46%)	6 (33%)	10 (71%)	9 (29%)	52 (39%)
Other	132	23 (100%)	44 (96%)	18 (100%)	14 (100%)	28 (90%)	127 (96%)

N, number of non-missing observations; n, number of hospitals; PIVC, peripheral intravenous catheter. ^a^Options not mutually exclusive. ^b^“No” category not shown. ^c^Nursing technicians require 20–24 months of training with a minimum high school education and nursing assistants require approximately 15 months of training with a minimum elementary school education.

Hospital practice regarding frequency of PIVC assessment varied, with hospitals in Argentina assessing the site every 6–8 h (8/13, 62%) and whenever the catheter was used (54%) (Table 1). Hospitals in Chile reported recommending PIVC assessment every 12–24 h (13/16, 81%); in Colombia, every 6–12 h (9/12, 75%); in Brazil, every 6 h (7/25, 28%) or whenever the catheter was used (8/25, 32%); and in Mexico eight hourly (16/23, 70%) (Table 1). The main reason for PIVC insertion was medications (7,732, 81%), followed by fluid therapy (5,676, 59%) (Table 2).

**TABLE 2:** Peripheral intravenous catheter insertion and assessment characteristics by country.

	*N*	Argentina	Brazil	Chile	Colombia	Mexico	Total
**Demographics**							
Number of hospitals	132	23 (17%)	46 (35%)	18 (14%)	14 (11%)	31 (23%)	132 (100%)
Number of PIVCs	9,620	947 (10%)	2,763 (29%)	1,431 (15%)	2,029 (21%)	2,450 (25%)	9,620 (100%)
Age (≥ 18 years)	9,330	839 (90%)	2,337 (86%)	1,204 (85%)	1,746 (90%)	1,933 (83%)	8,059 (86%)
Age [years; adults only; mean (SD)]	8,059	55.3 (20.3)	54.7 (19.1)	57.6 (19.8)	59.8 (19.9)	49.8 (18.1)	55.1 (19.6)
Male gender	9,619	536 (57%)	1,378 (50%)	754 (53%)	1,048 (52%)	1,191 (49%)	4,907 (51%)
**Assessment/selection**							
**Reason for insertion^a,b^**		***n* = 946**	***n* = 2,752**	***n* = 1,429**	***n* = 2,027**	***n* = 2,450**	
Intravenous medications	9,604	786 (83%)	2,406 (87%)	1,058 (74%)	1,548 (76%)	1,934 (79%)	7,732 (81%)
Intravenous fluids	9,604	739 (78%)	1,086 (39%)	765 (54%)	1,115 (55%)	1,971 (80%)	5,676 (59%)
Taking blood	9,604	8 (1%)	100 (4%)	103 (7%)	27 (1%)	175 (7%)	413 (4%)
Blood transfusion	9,604	28 (3%)	66 (2%)	13 (1%)	18 (1%)	163 (7%)	288 (3%)
**Puncture site^b^**	**9,359**	***n* = 936**	***n* = 2,653**	***n* = 1,394**	***n* = 1,966**	***n* = 2,410**	
Forearm		464 (50%)	1,012 (38%)	601 (43%)	780 (40%)	784 (33%)	3,641 (39%)
Hand		176 (19%)	696 (26%)	372 (27%)	645 (33%)	824 (34%)	2,713 (29%)
Wrist		109 (12%)	212 (8%)	134 (10%)	266 (14%)	397 (16%)	1,118 (12%)
Antecubital fossa		80 (9%)	516 (19%)	131 (9%)	129 (7%)	146 (6%)	1,002 (11%)
Arm		107 (11%)	217 (8%)	156 (11%)	146 (7%)	259 (11%)	885 (9%)
**Catheter type**	**9,613**	***n* = 946**	***n* = 2,761**	***n* = 1,429**	***n* = 2,027**	***n* = 2,450**	
Non-winged/non-ported		795 (84%)	2,508 (91%)	1,384 (97%)	1,953 (96%)	2,181 (89%)	8,821 (92%)
Ported		48 (5%)	50 (2%)	2 (< 1%)	29 (1%)	112 (5%)	241 (3%)
Plastic-winged with extension		58 (6%)	96 (3%)	21 (1%)	35 (2%)	3 (< 1%)	213 (2%)
Other		45 (5%)	107 (4%)	22 (2%)	10 (< 1%)	154 (6%)	338 (4%)
**Catheter size/gauge**	**9,100**	***n* = 802**	***n* = 2,507**	***n* = 1,359**	***n* = 1,989**	***n* = 2,443**	
18 gauge or larger		192 (24%)	342 (14%)	279 (21%)	429 (22%)	818 (33%)	2,060 (23%)
20–22 gauge		566 (71%)	1,836 (73%)	1,000 (74%)	1,410 (71%)	1,349 (55%)	6,161 (68%)
24 gauge or smaller		44 (5%)	329 (13%)	80 (6%)	150 (8%)	276 (11%)	879 (10%)
**Insertion**							
**Inserted by**	**9,083**	***n* = 915**	***n* = 2,463**	***n* = 1,308**	***n* = 1,968**	***n* = 2,429**	
Registered nurse		829 (91%)	289 (12%)	1,085 (83%)	281 (14%)	2,270 (93%)	4,754 (52%)
Nursing technician		48 (5%)	1,796 (73%)	9 (1%)	2 (<1%)	1 (<1%)	1,856 (20%)
Nursing assistant		10 (1%)	250 (10%)	0 (0%)	1,667 (85%)	24 (1%)	1,951 (21%)
Other		28 (3%)	128 (5%)	214 (16%)	18 (1%)	134 (6%)	522 (6%)
**Insertion location^b^**	**9,188**	***n* = 905**	***n* = 2,553**	***n* = 1,351**	***n* = 1,981**	***n* = 2,398**	
Medical/surgical wards		579 (64%)	1,543 (60%)	778 (58%)	945 (48%)	1,531 (64%)	5,376 (59%)
Emergency department		131 (14%)	466 (18%)	264 (20%)	720 (36%)	662 (27%)	2,243 (24%)
Operating room		124 (14%)	252 (10%)	204 (15%)	124 (6%)	94 (4%)	798 (9%)
Intensive/critical care unit		71 (8%)	292 (11%)	105 (8%)	192 (10%)	111 (5%)	771 (8%)
**Number of insertion attempts**	**7,737**	***n* = 753**	***n* = 2,024**	***n* = 981**	***n* = 1,701**	***n* = 2,278**	
1		557 (74%)	1,413 (70%)	758 (77%)	1,437 (84%)	1,728 (76%)	5,893 (76%)
2		124 (16%)	325 (16%)	139 (14%)	185 (11%)	390 (17%)	1,163 (15%)
3 or more		72 (10%)	286 (14%)	84 (9%)	79 (5%)	160 (7%)	681 (9%)
**Dressing**	**9,615**	***n* = 946**	***n* = 2,763**	***n* = 1,429**	***n* = 2,027**	***n* = 2,450**	
Simple transparent polyurethane		486 (51%)	629 (23%)	625 (44%)	334 (16%)	1,409 (58%)	3,483 (36%)
Bordered transparent polyurethane		148 (16%)	248 (9%)	510 (36%)	1,231 (61%)	859 (35%)	2,996 (31%)
Non-sterile tape only		159 (17%)	1,275 (46%)	7 (< 1%)	384 (19%)	25 (1%)	1,850 (19%)
Sterile gauze and tape		147 (16%)	64 (2%)	274 (19%)	4 (< 1%)	2 (< 1%)	491 (5%)
Other		6 (1%)	547 (20%)	13 (1%)	74 (4%)	155 (6%)	795 (8%)
**Management^c^**							
Dwell time [days; median (IQR)]	8,614	1 (1–3)	1 (0–2)	1 (1–2)	1 (1–3)	1 (1–3)	1 (1–2)
Assessment documented in previous 24 h^c^	9,608	322/946 (34%)	1,418/2,756 (51%)	1,017/1,429 (71%)	1,892/2,027 (93%)	1,584/2,450 (65%)	6,233 (65%)
Device not used in previous 24 h^c^	9,614	84/946 (9%)	349/2,762 (13%)	193/1,429 (14%)	98/2,027 (5%)	124/2,450 (5%)	848 (9%)
**Dressing integrity**		***n* = 946**	***n* = 2,754**	***n* = 1,429**	***n* = 2,027**	***n* = 2,450**	
Dressing clean, dry, and intact	9,606	778 (82%)	2,286 (83%)	1,217 (85%)	1,824 (90%)	2,190 (89%)	8,295 (86%)
Dressing moist, or soiled	9,606	33 (3%)	91 (3%)	39 (3%)	40 (2%)	68 (3%)	271 (3%)
Dry and soiled/stained	9,606	56 (6%)	139 (5%)	69 (5%)	57 (3%)	75 (3%)	369 (4%)
**PIVC complications**		***n* = 946**	***n* = 2,741**	***n* = 1,429**	***n* = 2,027**	***n* = 2,450**	
Blood in extension tubing/connection	9,593	111 (12%)	161 (6%)	96 (7%)	69 (3%)	132 (5%)	569 (6%)
Pain including on palpation	9,593	51 (5%)	148 (5%)	98 (7%)	95 (5%)	226 (9%)	618 (6%)
Dried blood around PIVC	9,593	38 (4%)	72 (3%)	55 (4%)	80 (4%)	76 (3%)	321 (3%)
Hyperemia > 1 cm from insertion	9,593	12 (1%)	42 (2%)	22 (2%)	24 (1%)	46 (2%)	146 (2%)
Edema > 1 cm from insertion	9,593	13 (1%)	34 (1%)	11 (1%)	9 (<1%)	36 (1%)	103 (1%)

24 h, 24 hours; IQR, intra-quartile range; N, number of non-missing observations; n, number of hospitals; PIVC, peripheral intravenous catheter; SD, standard deviation. ^a^Options not mutually exclusive. ^b^Options with <3% prevalence not shown. ^c^Frequency/number of hospitals (percentage) shown.

### PIVC inserter characteristics

PIVC insertion in all countries was predominantly performed by RNs (4,754, 52%) with a university degree, followed by nursing technicians (1,856, 20%) who generally have a secondary school education, receive up 15 months of training, and work independently of RNs in most cases. Nursing assistants inserted 21% (1,951) of all PIVCs. While their scope of practice varied between participating countries, nursing assistants may only have an elementary-level education, receive up to 12 months of clinical training, and usually undertake repetitive clinical activities under the direct supervision of an RN (Table 2).

PIVC insertion occurred primary in medical/surgical wards (5,376, 59%), followed by the emergency department (2,243, 24%), with most insertions successful on the first attempt (5,893, 76%) for RNs (75%), nursing technicians (72%), and particularly nursing assistants (83%). Colombian hospitals recorded the highest success rate for first-attempt insertion (1,437, 84%). Most PIVCs were secured with transparent polyurethane (simple 3,483, 36% or bordered 2,996, 31%); however, almost one-fifth (1,850, 19%) were dressed only with non-sterile tape, with hospitals in Brazil most often (1,275, 46%) using this method.

### Prevalence of PIVC complications

Audit data identified 14% (1,311) of PIVC dressings in all countries were not clean, dry, or intact, with blood in the extension tubing/connection (569, 6%) and dried blood around the PIVC site (321, 3%) also reported. The most common PIVC site complication was pain (618, 6%) (Table 2). Eight percent (745) of insertions had pain, hyperemia, or edema.

### Prevalence of redundant/Idle PIVCs

While the average PIVC dwell time at the time of observation was 1 day (IQR 1–2), 9% (848) of PIVCs were not used in the previous 24 h. However, PIVC assessment was documented in the previous 24 h in 65% of cases (6,233). Primarily, puncture sites were the forearm (3,641, 39%), hand (2,713, 29%), wrist (1,118, 12%), and antecubital fossa (1,002, 11%). Only Mexico had more insertions in the hand than forearm. Most catheters were 20–22 gauge (6,161, 68%), non-winged/non-ported catheters (8,821, 92%) inserted into adult patients (Table 2). By inserter group, doctors inserted catheters into the hand, wrist or antecubital fossa in 62% of cases, followed by nursing technicians (52%), nursing assistants (50%), RNs (49%), and IV team (48%).

Documentation related to insertion date, reason for insertion, and site assessment was absent in 52% of insertions by nursing technicians, 41% for RNs, and 14% of nursing assistants overall, with country-specific data reported in Table 3.

**TABLE 3:** Missing PIVC documentation (defined as missing insertion date, reason for insertion, or no evidence of site assessment in 24 h) by inserter (%).

Inserter	Argentina	Brazil	Chile	Colombia	Mexico
Registered nurses	69%	50%	29%	9%	39%
Nursing technicians	65%	51%	56%	0%	0%
Nursing assistants	80%	45%	NA	9%	25%

NA, not applicable.

## 4. Discussion

This practice evaluation surveyed hospitals from five Latin American countries regarding hospital PIVC policies and processes for PIVC insertion and maintenance, inserter characteristics, prevalence of PIVC complications, and redundant/idle PIVCs in a 24-h period. Key results are discussed as per the VHP quadrants.

### Hospital policies and processes for PIVC insertion and maintenance

Most hospitals recommended aseptic non-touch technique (69%) or strict asepsis (17%) for PIVC insertion. Mexican hospitals had a higher frequency of strict asepsis where sterile gloves were used (48%). While the use of sterile gloves has significant cost implications, there appears to be a strong belief that their use prevents PIVC infection even in the absence of high-level evidence (24). However, 14% of hospitals recommended a clean technique for PIVC insertion, which is not supported by recent clinical guidelines (25). Socio-economic barriers may contribute to some of the findings. For example, a lack of access to polyurethane dressings in some regions might have led to the use of non-sterile tape as a primary dressing, which contravenes best practice and increases the risk of bloodstream infection. Regardless, non-sterile tape was recommended as a primary dressing in 69% of hospitals, contravening internationally accepted clinical guidelines (24, 25). In addition, the recommended use of povidone-iodine in alcohol by 98% of participating hospitals contravenes randomized controlled trial results recently reported by Guenezan and colleagues, who found infections were significantly reduced with chlorhexidine and alcohol over povidone-iodine in alcohol (26). The Australian Clinical Care Standard for the management of PIVCs recommends the use of chlorhexidine 2% in 70% alcohol unless contraindicated (25), a recommendation made by 59% of hospitals.

International guidelines (24, 27, 28), a Cochrane systematic review (29), and results from randomized controlled trials undertaken in developed and developing countries, including Brazil (30–33), support clinically indicated PIVC replacement in adult patients. However, this was recommended by only 18% of participating hospitals, with significant variation in Brazilian (5%) and Colombian (36%) facilities. In addition, 22% reported very frequent replacement durations of 8–72 h, suggesting many unnecessary and unpleasant insertion procedures are still occurring (29). Furthermore, clinically indicated PIVC replacement is identified as standard practice across the literature for neonates and children (24). However, 41% of participating hospitals did not recommend this approach for pediatrics, suggesting barriers to effective and timely implementation of updated evidence and guidelines.

### PIVC inserter characteristics

For most PIVCs (93%), the inserter was a nurse, with only 2% inserted by doctors. Compared internationally, this is higher than in other continents and countries where nurses are the primary inserters in 71% of cases, as reported in a cross-sectional study of international PIVC practices (1). This worldwide phenomenon indicates PIVC insertion is predominately a nurse-led practice requiring specialized knowledge and core nursing skills. Our audit indicated PIVC insertions were successful on the first attempt in 76% of cases, which compares with reported rates of 86% in the emergency department (34). Nevertheless, this could be improved. Of concern, three or more insertion attempts were reported in 9% of cases, with Brazil reporting the highest proportion (14%).

Within the clinician cohort, 41% of PIVC insertions were by nursing technicians or assistants who often have less education than RNs and may have difficulties understanding hospital guidelines and procedures related to PIVC practice, particularly when this information is in English, the language in which most PIVC clinical guidelines are published (24, 27, 28, 35). However, their overall success rate for first-time insertions was consistently high, although we do not know if they had similar competence in all aspects of post-insertion care. Similar to other workforce models such as in the United States, assistants and technicians play a vital role in the Latin American healthcare context, including most aspects of healthcare delivery including PIVC insertion. However, some countries rely more heavily on nursing technicians and assistants for PIVC insertion, particularly in Brazil (73% of insertions by nursing technicians) and in Colombia (85% of insertions by nursing assistants). Currently, there is limited evidence that PIVC insertion undertaken by specialist nurse-led teams and advanced nurse practitioners has better clinical outcomes than those inserted by generalist groups of nurses, doctors, or other designated healthcare professionals (36–38).

Given nursing technicians and assistants represent a significant proportion of healthcare workers responsible for PIVC insertion and management, limited English language proficiency may contribute to knowledge deficits and delays in implementation of new techniques and evidence into clinical practice. As characterized by missing data (of insertion date, reason for insertion, or no documented site assessment in 24 h) in our audit, documentation of clinical data was absent or incomplete in 52% of insertions by nursing technicians but only 14% of insertions by nursing assistants. This suggests nursing assistants may be better at documenting PIVC cares, given that is one of their primary repetitive tasks, or it may reflect that most nursing assistant insertions occurred in Colombia where incidence of missing data entry was low for both assistant and RN insertions. Our results highlight the variety of workforce models and reinforce the importance of ongoing education and research into nursing workforce models in Latin American hospitals.

### Prevalence of PIVC complications

Patient-reported pain and blood in the extension tubing or connection were the most frequently reported adverse events (6% for each), followed by blood around the insertion site (3%). A recent systematic review of 103 prospective studies (76,977 PIVCs) reported a similar (6.4%) incidence of pain but did not include blood backflow nor site ooze (9). Blood backflow in tubing is an infection and occlusion risk and may have been related to use of non-blood control PIVCs as well as inadequate attention to positive pressure flushing practices in line with the INS Standard 41 “Flushing and Locking” (24). Blood at the insertion site is an infection risk and may have reflected difficult or poor technique insertions.

The majority (67%) of insertion sites were secured with sterile transparent film. However, many PIVCs (19%) were affixed with only non-sterile adhesive tape (no dressing), which increases the risk of infection, dislodgement, and patient discomfort (39). It is noteworthy that in Brazil this finding was even more prevalent (46%), despite Brazilian standards recommending non-sterile tape not to be used for PIVC stabilization or coverage (40). The use of non-sterile approaches to skin preparation and to stabilize/cover insertion sites must be eradicated to reduce the possibility of severe adverse events such as PIVC-associated bloodstream infection. Therefore, regular education for clinical staff should focus on dressing and securement selection, and management for infection prevention (39).

PIVC insertion in non-recommended sites such as the hand, antecubital fossa, and wrist occurred in 52% of patients, compared to 71% of patients reported by Alexandrou and colleagues (1). Doctors represented the workforce group that inserted the greatest number of PIVCs into non-recommended sites (62%), whereas RNs and intravenous teams had the lowest, with 49 and 48%, respectively. Insertion of PIVCs in sites of flexion increases the risk of subsequent complications like nerve injuries and phlebitis (24, 41, 42). As such, the forearm should be promoted as the ideal insertion site to prevent complications and improve patient comfort (24).

These findings suggest deficits in site assessment, device function, dressing and securement, as outlined in the VHP model (20, 21). Functions to ensure VHP include performing a visual assessment to evaluate the need to flush the line, or change the dressing using aseptic non-touch technique (20). Catheter patency is achieved by gentle, periodic flushing of the line with 0.9% sodium chloride solution, but recent studies have highlighted a lack of nursing knowledge and subsequent inconsistencies in this practice (43–45). Other complications, such as pain, may have resulted from poor vein choice and insertion into sites of flexion by the inserter (20).

In comparison with our findings, in a randomized clinical trial conducted in Brazil between 2012 and 2015 (*n* = 169 adults), the overall proportion of PIVC complications was 55% (*n* = 94), resulting from phlebitis, infiltration, obstruction, and dislodgement (46). An earlier 2010 prospective cohort study (*n* = 198 adults) by Colombian researchers found a cumulative incidence of phlebitis per catheter of 10% [*n* = 20; 95% confidence interval (CI) 6–15%], with phlebitis representing 53% (*n* = 17) of all PIVC complications (47). In 2015, Mexican researchers reported 47/211 (22%) of pediatric patients with PIVCs had chemical phlebitis (48). Phlebitis was also a problem in a 2014 Chilean study whose authors reported 196 cases of phlebitis in adult and pediatric patients (participant numbers not reported, with overall incidence rate of phlebitis 2.4 per 1,000 bed-days) with PIVCs over a 6-month period; of these, 10 were infectious (5 associated with bacteremia and 5 had local purulent discharge) and 186 non-infectious, appearing on average 2.5 days after PIVC insertion (range 0–17 days) (49).

### Prevalence of redundant/Idle PIVCs

Failure to routinely assess and document PIVCs can lead to idle catheters, missed care, and potential for consequences such as bloodstream infection (50). Results indicated 9% of PIVCs had not been used in the previous 24 h, with Chile having the highest rate of redundancy (14%), and Colombia and Mexico the lowest (5%). While incidences were equal or lower than the 14% reported by Alexandrou et al. for PIVCs observed in 2014–2015 (1), they may indicate that the increasing adoption of clinically indicated removal policies are driving down idle catheters in the region.

Routine PIVC assessment should include daily re-evaluation of device necessity, and when treatment is deemed to be complete, the device should be immediately removed to avoid complications (50). Documentation of PIVC site assessment in the previous 24 h was reported in only 51% by Alexandrou et al. (1), but in our study was 65% of PIVCs overall, with Colombia having the highest (93%) and Argentina the lowest (34%) levels. Comprehensive and timely documentation ensures consistent and accurate information across the care continuum (51). Accurate and complete nursing records are essential for monitoring health indicators to ensure patient safety (52) and cost efficiencies (53). Therefore, PIVC education should stress the importance of documentation in achieving patient safety and reducing adverse incidents.

### Recommendations

The development of multinational communities of practice to promote and regularly review current vascular access practices (using regular audit, surveillance, and patient satisfaction measures) are recommended. Some participating countries already have well developed vascular access societies that could be used to model for other countries in Latin America. These could work together to identify practice deficits and complications, and to tailor education programs for clinicians responsible for PIVC insertion based on the latest evidence and guidelines.

The use of PIVCs across the globe and the impact on crucial hospital indicators, such as patient experience, nursing satisfaction with the practice environment, and potentially harmful and costly adverse events justifies national and local investments to achieve better outcomes, prioritizing consistent application of good clinical practice. We believe our results provide several areas for quality and process improvements, namely: (1) lower tolerance for painful catheters, which should include strategies to prevent pain and prompt removal when pain is evident; (2) investment to trial technologies that avoid blood backflow into PIVCs and tubing/connectors, paired in the short term with improved nursing attention to flushing of visible blood; (3) improved inserter competence to avoid difficult insertions, with subsequent cleaning of dried blood from insertion sites when necessary, or use of tissue adhesive for patients prone to blood ooze; (4) update of insertion policies to preferentially decontaminate skin with alcoholic chlorhexidine solution, and removal policies to clinically indicated criteria; (5) de-implementation of non-sterile tape as a dressing; and (6) ongoing campaigns to promote regular (minimum daily) documented site assessment and prompt removal of idle catheters.

To achieve this, evidence-based vascular access and infection prevention guidelines must be made available by government and professional organizations in Latin America in languages other than English. Without regular evidence updates available in Spanish and Portuguese, it is extremely difficult to expect Latin American nurses and nursing assistants to be adhering to evidence-based practice in vascular access device management. Next, PIVC insertion education should promote site assessment and selection, with inserters taught the principles of VHP, such as choosing the appropriate device for the individual patient and medical treatment, and avoiding areas of flexion, if possible. Each health service should have evidence of a locally approved policy that ensures healthcare professionals are competent in PIVC insertion, monitoring, and removal. In line with existing Standards (24), the policy should specify the: competency a clinician must demonstrate to insert a PIVC, including for more complex and technology-assisted insertions; competency a clinician must demonstrate to monitor and remove PIVCs; the organizational process to assess and monitor the ongoing competency of clinicians, including for more complex insertions; and, the organizational process to assess adherence to the policy. Further, the Association for Vascular Access offers a free, comprehensive PIVC curriculum to universities that is currently available only in English^1^ ; we call for translation and availability of this curriculum for LATAM. Maintenance education should focus on daily review of device necessity and prompt removal of idle PIVCs, as well as the importance of routinely checking patency. As an urgent infection-prevention measure, non-sterile tape should be discontinued for use as a primary dressing, as well as improved nurse decision-making to replace dressings at any point that are not clean, dry, and intact. Finally, documentation of PIVC insertion and routine assessment should be considered a patient safety priority.

We found the VHP model particularly helpful in structuring the data analysis and in identifying hospital policies and processes for PIVC insertion and maintenance and reporting prevalence audits according to the PIVC selection, insertion, management, and evaluation processes. When used in combination, VHP quadrant functions may contribute to a wider program of PIVC evidence-based quality and patient safety practice. We therefore recommend that hospital quality improvement and patient safety initiatives follow this model for structuring PIVC education and reporting audit results.

### Strengths and limitations

A strength of our study was the multi-country, stratified sampling approach that allowed in-depth understanding of PIVC evidence-based care in Latin America. This enabled a broader scope of hospitals that included different regions and types of hospitals. This study expanded upon the work undertaken in the “One Million Global Catheters” study that focused mainly on metropolitan hospitals that were often better-funded, academic centers (1). AIs enabled adaption to patient and organizational contexts with emphasis on social and cultural norms, ensured standardization, translation, and appropriate application of the data collection instruments. The main limitations to this study included lack of resources to recruit in all countries in line with our sampling frame, particularly in remote hospitals, lack of time for collectors to collect data due to staff shortages, and heavy workloads. This may have constituted selection bias and our results may not be generalizable to all hospitals in the region. In addition, the cross-sectional design meant that we could only identify prevalence of complications or poor practices at one time-point during PIVC dwell, thus our results likely greatly underestimate the incidence of complications per catheter.

### Impact on clinical practice

This study contributes to the identification of important factors such as level of education and access to evidence-based clinical guidelines that may hinder nursing practice for patients with a PIVC, with consequences for safety and quality. The results identify areas of fragility such as continued use of outdated and potentially harmful PIVC practices in participating countries, such as the use of clean over aseptic technique for PIVC insertion, use of povidone-iodine as a cleaning solution, insertion into non-recommended sites such as the hand, antecubital fossa, and wrist, dressing PIVC sites with non-sterile tape, and maintenance of redundant PIVCs. These highlight the importance of providing language-appropriate, updated evidence-based guidelines and PIVC education to nurses at the point of care, with periodic audits to ensure compliance with recommendations. Future research should examine longitudinal outcomes for patients with a PIVC and cost-effectiveness of the materials used in Latin American health institutions.

## 5. Conclusion

The enthusiastic support for this study from 132 Latin American hospitals demonstrates a willingness to engage in research and improve clinical practice. This is an opportunity for existing and successful vascular access societies to expand their reach and encourage other countries to join in multinational communities of practice. This study identified several outdated practices (including povidone-iodine cleaning solutions and non-sterile tape PIVC fixation) prevalent in Latin American countries. To facilitate evidence implementation in practice, clinical guidelines should be available in languages other than English.

## Data availability statement

The raw data supporting the conclusions of this article will be made available by the authors, on request.

## Ethics statement

The study was approved by the Griffith University Human Research Ethics Committee (Ref No: 2018/292). Participating countries obtained ethics approvals according to their hospital, regional or national requirements, and all participants or their guardians provided informed written consent. Written informed consent to participate in this study was provided by the participants or their legal guardian/next of kin.

## Author contributions

RW, MPr, MPd, and CR conceived the study, designed the protocol, obtained ethical approval, contributed to the data collection and analysis, wrote, and revised the final manuscript. GR-B, MC, and GM contributed to the protocol design, provided content expertise, and contributed to the writing, and revision of the final manuscript. MPt, SA, and MF obtained ethical approval, contributed to the data collection and analysis and writing the final manuscript. CÁ, MQ, MCC, GV, and EC obtained ethical approval and contributed to data collection and the refinement of the manuscript. All authors contributed to the article and approved the submitted version.

## Footnotes

1
http://www.avainfo.org/page/piveducation
