# Supplementary material for: Peripheral vascular catheter use in Latin America (the vascular study): A multinational cross-sectional study
Source: Front Med (Lausanne). 2023 Jan 4;9:1039232. doi: 10.3389/fmed.2022.1039232 (PMC9846050; doi:10.3389/fmed.2022.1039232)
Supplement: Supplementary file 1 [file Data_Sheet_1.zip › Supplementary File 1.DOCX]

**SUPPLEMENTARY FILE 1 – SAMPLING FRAME**

*Study settings:* Hospitals from Argentina, Brazil, Chile, Colombia, and Mexico were recruited. The participating countries are the most densely populated in Latin America, with large urban populations.^(1, 2)^ The hospitals invited to participate in this study belonged to one of the four to five geopolitical regions defined per country. In the Latin American context ‘region’ does not necessarily refer to semi-rural areas, rather areas with definable characteristics.

A purposive sample included hospitals with public or private funding (or a mix of funding), stratified according to **population per region.** It was expected that each participating country would invite health services to participate based on the population of each region,^(3)^ with a probable recruitment of 50 (following a 30% attrition rate). Approximately 10,000 to 15,000 patients from a variety of health settings in each country were targeted be recruited. Refer to Tables 1 to 5 for the sample for each participating country based on the population per region.

| **Table 1 – Sample according to the number of hospitals in Argentina.** | | | |
| --- | --- | --- | --- |
| ***Regions*** | ***Population (millions)*** | ***%*** | ***Hospital sample (number)*** |
| Argentine Northwest | 3,946,781 | 10.1 | 3 |
| Gran Chaco | 2,594,552 | 6.6 | 1 |
| Mesopotamia (or Littoral) | 3,503,891 | 8.9 | 2 |
| Cuyo | 3,384,570 | 8.7 | 2 |
| Pampas | 23,457,202 | 59.9 | 14 |
| Patagonia | 2,271,863 | 5.8 | 1 |
| **Total** 100.0 | 39,158,859 | 100.0 | 23 |
| There are 2,268 [hospitals](https://en.wikipedia.org/wiki/Hospital) in [Argentina](https://en.wikipedia.org/wiki/Argentina).  Source: Census 2013  <https://en.wikipedia.org/wiki/List_of_Argentine_provinces_by_population>  <https://pt.wikipedia.org/wiki/Ficheiro:Regiones_de_Argentina.svg>  Number of hospitals in selected countries in Latin America as of July 2017 (<https://www.statista.com/statistics/801867/number-hospitals-latam/>) | | | |

| **Table 2– Sample according to the number of hospitals in Brazil** | | | |
| --- | --- | --- | --- |
| ***Regions*** | ***Population (millions)*** | ***%*** | ***Hospital sample (number)*** |
| North | 17,936,201 | 8.6 | 7 |
| Northeast | 57,254,159 | 27.6 | 23 |
| Southeast | 86,949,714 | 41.9 | 34 |
| South | 29,644,948 | 14.3 | 12 |
| Central-Western | 15,875,907 | 7.6 | 6 |
| Total | 207,660,929 | 100 | 82 |
| There are 8,211 [hospitals](https://en.wikipedia.org/wiki/Hospital) in Brazil.  Source: IBGE. Diretoria de Pesquisas - DPE – Coordenação de População e Indicadores Sociais - COPIS. (<ftp://ftp.ibge.gov.br/Estimativas_de_Populacao/Estimativas_2017/estimativa_dou_2017.pdf>)  Number of hospitals in selected countries in Latin America as of July 2017 (<https://www.statista.com/statistics/801867/number-hospitals-latam/>) | | | |

| **Table 3 – Sample according to the number of hospitals in Chile** | | | |
| --- | --- | --- | --- |
| ***Regions*** | ***Population (millions)*** | ***%*** | ***Hospital sample (number)*** |
| Norte | 1,485,438 | 8.3 | 1 |
| Centro | 11,742,259 | 65.8 | 3 |
| Sur | 4,591,357 | 25.9 | 2 |
| **Total** | 17,819,054 | 100.0 | 6 |
| There are 535 hospitals in Chile.  Source: DEIS Minsal 2018  [http://www.deis.cl/wp-content/uploads/2015/05/Análisis-de-situación-nuevas-proyecciones-de-población-INE.pdf](http://www.deis.cl/wp-content/uploads/2015/05/An%C3%A1lisis-de-situaci%C3%B3n-nuevas-proyecciones-de-poblaci%C3%B3n-INE.pdf)  Number of hospitals in selected countries in Latin America as of October 2017 (http://www.ine.cl/docs/default-source/publicaciones/2017/compendio-estadistico-2017.pdf?sfvrsn=6) | | | |

| **Table 4 – Sample according to the number of hospitals in Colombia** | | | |
| --- | --- | --- | --- |
| ***Regions*** | ***Population (millions)*** | ***%*** | ***Hospital sample (number)*** |
| Andean | 28,863,217 | 69.9 | 17 |
| Caribbean | 9,506,220 | 22.9 | 5 |
| Pacific | 1,078,753 | 2.6 | 1 |
| Orinoquía | 1,681,273 | 4.1 | 1 |
| Amazon | 264,945 | 0.6 | 1 |
| Insular | 74,620 | 0.2 |  |
| Total | 41,469,028 | 100.0 | 25 |
| There are 2,458 [hospitals](https://en.wikipedia.org/wiki/Hospital) in Colombia.  Source: Census 2005*  <https://en.wikipedia.org/wiki/List_of_Colombian_Departments_by_population>  <https://en.wikipedia.org/wiki/Natural_regions_of_Colombia>  <https://es.wikipedia.org/wiki/Regiones_naturales_de_Colombia>  <https://es.wikipedia.org/wiki/Demograf%C3%ADa_de_Colombia>  ***NB** Although there is a more current Census, the number of population by region was not found, because this calculation is not based on the number of population of the state, since a state may have belong to more than one region. Thus, the approximate values of 2005 were used as reference since most of the regions refer to this year of investigation (Census 2005 - Population Total: 41,468,384).  Number of hospitals in selected countries in Latin America as of July 2017 (<https://www.statista.com/statistics/801867/number-hospitals-latam/>) | | | |

| **Table 5 – Sample according to the number of hospitals in Mexico** | | | |
| --- | --- | --- | --- |
| ***Regions*** | ***Population (millions)*** | ***%*** | ***Hospital sample (number)*** |
| Northwest | 15,155,774 | 13.7 | 5 |
| Northeast | 11,516,117 | 10.4 | 4 |
| North-Central | 13,501,622 | 12.2 | 4 |
| South-Central | 18,091,419 | 16.4 | 6 |
| West | 14,321,586 | 13.0 | 5 |
| East | 18,412,594 | 16.6 | 6 |
| Southeast | 6,893,940 | 6.2 | 3 |
| Southwest | 12,719,048 | 11.5 | 4 |
| **Total** | 110,612,100 | 100.0 | 37 |
| There are 3,642 hospitals in Mexico.  Source: Census 2015  <https://en.wikipedia.org/wiki/List_of_Mexican_states_by_population>  <https://pt.wikipedia.org/wiki/Ficheiro:Regions_of_Mexico.svg>  Number of hospitals in selected countries in Latin America as of July 2017 (<https://www.statista.com/statistics/801867/number-hospitals-latam/>) | | | |

### **References**

1. Braga GB, Remoaldo PC, Fiúza ALdC. A methodology for definition of rural spaces: an implementation in Brazil. Ciência Rural. 2016;46:375-80.

2. Review WP. Latin America Population2017; 2018(March 12). Available from: <http://worldpopulationreview.com/continents/latin-america-population/>.

3. Alexandrou E, Ray-Barruel G, Carr PJ, Frost S, Inwood S, Higgins N, et al. International prevalence of the use of peripheral intravenous catheters. Journal of hospital medicine. 2015;10(8):530-3.
