# Supplementary material for: Peripheral vascular catheter use in Latin America (the vascular study): A multinational cross-sectional study
Source: Front Med (Lausanne). 2023 Jan 4;9:1039232. doi: 10.3389/fmed.2022.1039232 (PMC9846050; doi:10.3389/fmed.2022.1039232)
Supplement: Supplementary file 1 [file Data_Sheet_1.zip › Supplementary File 2.PDF]

VASCULAR Study: Frequently Asked Questions (FAQs)

Why is the study important?

The study is designed to discover what is actually happening with PIVCs in clinical practice. Are research findings used to improve health care? Are we doing the best we can for our patients?

We believe the study will help people to evaluate their own practice, compare it to the research, benchmark with other hospitals, and discover ways to improve patient safety and healthcare.

This study of PIVCs in Latin America will open up new collaborative research opportunities in vascular access clinical practice.

What date is the study scheduled for?

The choice of date for the study can be decided by the individual organisation, depending on staffing, workload and other local issues. We suggest that each site choose a day in 2018 to complete the study. We will collect data until December 2018.

We recommend that a group of assessors (nurse educator, clinical nurse specialist, vascular access clinician, etc) be educated about the study and the data collection tools. Each assessor can then assess the PIVCs on 2 or 3 wards/units, rather than a 2 or 3 staff trying to collect data for the whole hospital.

It might be more feasible for your site to collect data over a week, rather than a single day. This is fine.

What forms do we need to complete?

1. The **Control form** is for each country/region to identify the data collection methods (paper or electronic), the proposed date of data collection, the completion of data collection and the receipt of completed paper or electronic data,
2. The **Site Information form** is filled in once per hospital.
3. The **Screening log** will be filled in on the day of data collection by each participating ward/unit.
4. The **Data Collection form** will be completed for every PIVC in situ on the ward/unit at time of data collection.

- All of the study tools will be available for completion on paper or on-line.
- A survey link will be emailed to you to complete the forms electronically.
- If you choose to complete the study on paper, you can then enter the data via the survey link.
- Alternatively, you can fax or scan and email the completed forms to or contact us for postage details.

How long will it take to assess each PIVC?

Feedback from the OMG pilot study indicated it took approximately 5 minutes to assess the first few PIVCs, but became quicker as the assessor became more experienced with the tools.

The tools have been modified since the pilot study and this will hopefully now be more streamlined.

Do we need Human Research Ethics Committee (HREC) or Institutional Review Board (IRB) approval for our site to participate in the study?

The study has been approved by the Griffith University Human Research Ethics Committee *GU Ref No: 2018/292.*

Approval on a site-specific basis is an individual matter. Many sites have not required site-specific ethics approval because this is an observational audit, rather than an intervention study. Other sites have requested approval. We can provide some assistance with obtaining ethical approval, if required.

Please note: Some peer-reviewed journals may request notification that the IRB has been informed of the study and have advised that IRB approval is not required.

Are there benefits for participating organisations?

There are certainly benefits for participating organisations. These include:

- Benchmarking opportunities with other organisations in your region or country
- Potential for identifying quality assurance concerns at your organisation, such as use of consumables (dressings, IV cannulas, etc.), number of redundant catheters, etc.
- Potential for developing targeted education programs to address areas of concern, such as non-compliance with recommended guidelines, IV dressing techniques, etc.
- Potential for publication of site-specific findings.

What will happen to our data?

Each hospital in the study will be de-identified and have a unique identifier. We will not share your data with other organisations. We will publish the results by country, not by hospital. Each participating site will be acknowledged as a contributor to the study, but individual hospital results will remain confidential. Any information you collect remains the property of your hospital.

All data will be stored securely at Griffith University Centre for Health Practice Innovation. Only the VASCULAR study investigators will have access to the data. All stored data will be destroyed after 7 years, as per the Human Research Ethics Committee requirement.

Will we be benchmarked against similar organisations?

For best benchmarking with other hospitals in your region, it would be ideal if all local sites could complete on the same day or in the same week. This can be coordinated locally.

We will provide every participating organisation with a copy of its own results. You can use this information to benchmark your results with other hospitals in your local region or country.

Do we need to assess every PIVC?

The more data you are able to collect, the better will be your opportunities for benchmarking, but we do understand that for many reasons it may not be possible to assess every PIVC.

What about patients that cannot give verbal consent? (e.g., paediatrics, unconscious or sedated patients)

If the patient is not able to give informed verbal consent, please follow your institution's policy to determine if you can ask the patient's next-of-kin for consent.

Any patients who do not give consent will not have their PIVC assessed for the study.

What about incomplete data?

We understand that it may not be possible to obtain all the data on the form. If the data is not readily available (such as date and time of IV insertion), it is okay to ask the patient, if feasible.

For benchmarking purposes, more data is better, but realistically this may not be possible. This is fine.

Can we publish our own findings?

After the main study has been completed and results published in a peer-reviewed journal by the VASCULAR study team, there will also be the opportunity to publish your site-specific findings. We ask that you acknowledge the VASCULAR study team in any publications. An authorship agreement form is included in this study guide.

Are we permitted to see the results of the pilot study?

This data will be submitted for publication in a peer-reviewed journal. No identifying information will be presented. The results will be published per country. We will advise when this publication becomes available.

For any further questions, please email the Principal Chief Investigator Dr Rachel Walker at r.walker@griffith.edu.au

Estudio VASCULAR
Preguntas Frecuentes

¿Por qué es importante el estudio?

El estudio está diseñado para descubrir qué está sucediendo realmente con los catéteres venosos periféricos cortos CVPCs en la práctica clínica. ¿Los hallazgos de investigación se utilizan para mejorar la atención médica? ¿Estamos haciendo lo mejor que podemos por nuestros pacientes?

Creemos que el estudio ayudará a las personas a evaluar su propia práctica, compararla con la investigación, establecer un punto de referencia con otros hospitales y descubrir formas de mejorar la seguridad y la atención médica del paciente.

Este estudio de catéteres venosos periféricos cortos CVPCs en América Latina abrirá nuevas oportunidades de investigación en la práctica clínica de acceso vascular.

¿En qué fecha está programado el estudio?

La elección de la fecha para el estudio puede ser decidida por la organización individual, dependiendo del personal, la carga de trabajo y otros asuntos locales. Sugerimos que cada sitio elija un día en 2018 para completar el estudio. Recopilaremos datos hasta diciembre de 2018.

Recomendamos que un grupo de evaluadores (educador de enfermería, especialista en enfermería clínica, clínico de acceso vascular, etc.) se eduque sobre el estudio y las herramientas de recopilación de datos. Cada asesor puede evaluar los catéteres venosos periféricos cortos CVPCs en 2 ó 3 salas/unidades, en lugar de 2 ó 3 miembros del personal que intenta recopilar datos para todo el hospital.

Podría ser más factible que su sitio recopile datos durante una semana, en lugar de un solo día. Esto sería adecuado.

¿Qué formularios necesitamos completar?

1. El **Formulario de Control** es para que cada país/región identifique los métodos de recopilación de datos (en papel o electrónicos), la fecha propuesta para la recopilación de datos, la finalización de recopilación de datos y la recepción de los datos en papel o electrónicos ya diligenciados.
2. El **Formulario de Información del sitio** se completa una vez por hospital.
3. El **Registro de pacientes del servicio/unidad** se completará el día de la recopilación de datos por cada sala/unidad participante.
4. El **Formulario de Recolección de Datos** se completará por cada catéter venoso periférico corto CVPC canalizado en la sala/unidad en el momento de la recolección de datos.

- Todas las herramientas de estudio estarán disponibles para completar en papel u online.

- Se le enviará un enlace de la encuesta por correo electrónico para completar los formularios electrónicamente.
- Si elige completar el estudio en papel, puede ingresar los datos a través del enlace de la encuesta.
- Como alternativa, puede enviar por fax o escanear y enviar por correo electrónico los formularios completos a m.arneil@griffith.edu.au o contactarnos para detalles de envío con copia al correo vascularstudycolombia@gmail.com.

¿Cuánto tiempo llevará evaluar cada catéter venoso periférico corto CVPC?

Los comentarios del estudio piloto de OMG indicaron que tomó aproximadamente 5 minutos evaluar los primeros catéteres venosos periféricos cortos CVPCs, pero se hizo más rápido a medida que el evaluador adquirió más experiencia con las herramientas.

Las herramientas se han modificado desde el estudio piloto y, con un poco de suerte, esta será más ágil.

¿Necesitamos la aprobación del Comité de Ética en Investigación Humana (HREC) o la junta de Revisión Institucional (IRB) para que nuestro sitio participe en el estudio?

El estudio ha sido aprobado por el Comité de Ética en Investigación Humana de la Universidad de Griffith GU Ref No: 2018/292.

La aprobación en un sitio específico es un asunto individual. Muchos sitios no han requerido aprobación de ética específica del sitio porque esta es una auditoría observacional, en lugar de un estudio de intervención. Otros sitios han solicitado aprobación. Podemos proporcionar cierta asistencia para obtener la aprobación ética, si es necesario.

Tenga en cuenta: que algunas revistas revisadas por pares pueden solicitar una notificación de que la junta de Revisión Institucional (IRB) ha sido informado del estudio y han informado que no se requiere la aprobación del IRB.

¿Hay beneficios para las organizaciones participantes?

Sin duda, hay beneficios para las organizaciones participantes. Estas incluyen:

- Oportunidades de benchmarking con otras organizaciones en su región o país.
- Posibilidad de identificar problemas de garantía de calidad en su organización, como el uso de consumibles (vendajes, cánulas intravenosas, etc.), el número de catéteres innecesarios, etc.
- Potencial para desarrollar programas educativos específicos para abordar áreas de preocupación, como el incumplimiento de las guías recomendadas, técnicas de vendaje IV, etc.
- Posibilidad de publicación de hallazgos específicos del sitio.

¿Que pasará con nuestros datos?

Cada hospital del estudio estará sin identificación y se le asignara un código de identificación único. No compartiremos sus datos con otras organizaciones. Publicaremos los resultados

por país, no por hospital. Se reconocerá a cada sitio participante como colaborador del estudio, pero los resultados de cada hospital permanecerán confidenciales. Toda la información que recopile es propiedad de su hospital.

Todos los datos se almacenarán de forma segura en el Centro Universitario Griffith de Innovación en la Práctica de la Salud. Solo los investigadores del estudio VASCULAR tendrán acceso a los datos. Todos los datos almacenados se destruirán después de 7 años, según el requisito del Comité de ética de la investigación humana.

¿Seremos comparados con otras organizaciones similares?

Para una mejor evaluación comparativa con otros hospitales en su región, sería ideal si todos los sitios locales pudieran completarse el mismo día o en la misma semana. Esto puede ser coordinado localmente.

Proporcionaremos a cada organización participante una copia de sus propios resultados. Puede usar esta información para comparar sus resultados con otros hospitales en su país o región local.

¿Necesitamos evaluar cada catéter venoso periférico corto CVPC?

Cuantos más datos pueda recopilar, mejores serán sus oportunidades para la evaluación comparativa, pero entendemos que, por muchas razones, puede que no sea posible evaluar cada catéter venoso periférico corto CVPC.

¿Que pasa con los pacientes que no pueden dar su consentimiento verbal? (por ejemplo: pacientes pediátricos, inconscientes o sedados)

Si el paciente no puede dar su consentimiento verbal informado, siga la política de su institución para determinar si puede solicitar el consentimiento de los familiares del paciente.

A los pacientes que no den su consentimiento no se les evaluará su catéter venoso periférico corto CVPC para el estudio.

¿Qué hay de los datos incompletos?

Entendemos que puede que no sea posible obtener todos los datos en el formulario. Si los datos no están disponibles (como la fecha y la hora de la inserción IV), es correcto preguntar al paciente, si es posible.

Para fines de evaluación comparativa, es mejor obtener más datos, pero de manera realista esto puede no ser posible. Esto es aceptable.

¿Podemos publicar nuestros propios hallazgos?

Después de que el estudio principal haya sido completado y los resultados publicados en una revista revisada por pares por el equipo de estudio de VASCULAR, también habrá la oportunidad de publicar los hallazgos específicos de su sitio. Le pedimos que reconozca al equipo de estudio VASCULAR en cualquier publicación. Se incluye un formulario “de acuerdo de autoría” en esta guía de estudio.

¿Se nos permite ver los resultados del estudio piloto?

Estos datos se enviarán para su publicación en una revista revisada por pares. No se presentará información de identificación. Los resultados serán publicados por país. Le avisaremos cuando esta publicación esté disponible.

Para cualquier otra pregunta, por favor envíe un correo electrónico a la Investigadora Principal, Dra. Rachel Walker at r.walker@griffith.edu.au con copia a el correo vascularstudycolombia@gmail.com.

Informações sobre a pesquisa

Vascular AccesS Catheter Use in Latin AmeRica (VASCULAR study)
Uso de cateteres intravenosos periféricos na América Latina: Estudo VASCULAR

Pesquisador Principal da Griffith University: Dra. Rachel Walker
School of Nursing and Midwifery,
Griffith University, Brisbane, Australia
r.walker@griffith.edu.au

Pesquisadores da Griffith University: Profa. Dra. Claire Rickard
Dra. Marie Cooke
Dra. Gillian Ray-Barruel

Pesquisadores Principais da América Latina: Profa. Dra. Mavilde LG Pedreira
Escola Paulista de Enfermagem – EPE
Universidade Federal de São Paulo – Unifesp
São Paulo, Brasil.mpedreira@unifesp.br
Enf. Cirlia Alvarez – Argentina
Enf. Martha Claudia Corzo – Colômbia
Enf. Gabriela Cortês Villarreal – México
Dra. Marcela Quintanillas Reyes - Chile

Pesquisadores do Brasil: Profa. Dra. Mavilde LG Pedreira
Profa. Dra. Maria Paula de Oliveira Pires
Profa. Dra. Maria Angélica S. Peterlini
Profa. Dra. Maria de Jesus C. S. Harada
Profa. Dra. Denise M Kusahara
Dra. Patrícia Vendramin
Enfa. Marcelle di Angelis Ambar Felipe
Enfa. Silvia Schoenau de Azevedo

Por que esta pesquisa está sendo realizada?

O Estudo VASCULAR é uma pesquisa de prevalência sobre uso, avaliação e manuseio de cateteres intravenosos periféricos (CIP) em hospitais da América Latina. Este estudo irá fornecer dados previamente indisponíveis sobre a prevalência e manuseio de CIP na América Latina, incluindo a duração média de instalação de CIP e a identificação de fatores de risco para complicações relacionadas ao CIP. Tais informações são valiosas e podem potencialmente prevenir milhões de reinserções desnecessárias de CIP e reduzir substancialmente os custos relacionados, principalmente em países em desenvolvimento. O estudo irá também gerar informações importantes quanto à adoção de boas práticas no cuidado e manuseio de tais dispositivos. Em geral, as evidências obtidas com essa pesquisa poderão ser utilizadas no direcionamento de protocolos clínicos e políticas de assistência à saúde e também melhorar os resultados para os pacientes, quanto ao cuidado e ao manejo de CIP.

Objetivos e Finalidades do Estudo

1. Identificar e comparar a prevalência de CIP em hospitais da América Latina.
2. Identificar e comparar a prevalência de CIP desnecessários ou não utilizados em um período de 24 horas em hospitais da América Latina.
3. Avaliar e comparar a prevalência de complicações relacionadas ao CIP (extravasamento, infiltração, infecção, flebite, oclusão, dor, dentre outras) em hospitais da América Latina.
4. Identificar a prevalência de fatores de risco para infecção ou complicações no uso do CIP.
5. Identificar práticas vigentes sobre higienização das mãos e preparo da pele; seleção dos dispositivos de infusão; estabilização e curativos; técnicas para manutenção do CIP e compará-las com recomendações e diretrizes.
6. Fornecer evidências e recomendações para fortalecer e melhorar os programas de educação e auditoria de CIP.
7. Identificar grupos populacionais de risco para complicações relacionadas ao CIP e que necessitam de mediadas específicas de prevenção.
8. Contribuir para a avaliação de custos ocasionados pelas complicações na população de pacientes e nas unidades de saúde. Identificar áreas para futuras pesquisas e projetos-piloto para iniciativas de melhoria e programas de educação para enfermeiros e profissionais da área da saúde.
9. Avaliar e estimar os custos relacionados à punção intravenosa periférica em hospitais da América Latina.
10. Fornecer recomendações para a prática clínica, educação e pesquisas futuras.

Quando a coleta de dados do estudo será realizada? Quem irá coletar os dados?

Em um determinado dia, todas as instituições que derem anuência em participar do estudo, bem como os profissionais e pacientes seu consentimento, serão orientadas a realizar auditoria de uso de CIP. A coleta dos dados que ocorrerá no primeiro semestre de 2019.

O que você terá que fazer?

Profissionais que aceitarem participar do estudo serão convidados a preencher três tipos diferentes de formulários.

1- O **"Formulário de Informações da Instituição"**, preenchido uma única vez por instituição, contém perguntas sobre: quem é o responsável pela inserção dos CIP na sua instituição; quais recomendações são seguidas pela sua instituição quanto à inserção e manutenção dos CIP; material e marca dos CIP utilizados na sua instituição; curativos e coberturas de CIP utilizados na sua instituição; soluções de limpeza e antissépticas utilizadas na sua instituição para a inserção de CIP e troca de curativos.

2- Você será solicitado também a preencher o **"Formulário de Coleta de Dados"** sobre o CIP. As informações incluirão:

- Idade e sexo do paciente.
- Condição de saúde: clínica/ cirúrgica/oncológica/intensiva.
- Data e local da inserção do CIP.
- Tipo e marca do cateter.
- Responsável pela inserção do cateter.
- Ambiente onde a punção foi realizada.
- Sítio de inserção do CIP.
- Calibre do CIP.
- Dispositivos intravenosos utilizados.
- Condições do sítio de inserção do CIP.
- Método de estabilização do CIP.
- Tipo de curativo.
- Avaliação do curativo.
- Prescrição da terapia intravenosa.
- Fluidos e medicamentos administrados.

3- O **"Formulário de triagem do serviço/unidade"** poderá ser preenchido no dia da coleta dos dados segundo cada unidade participante, a fim de auxiliar no controle das informações coletadas, prevenindo duplicações de dados. Nele constam as seguintes informações: tipo de unidade; consentimento/assentimento do paciente e ou responsável; iniciais do avaliador responsável pela informação.

Todos os dados serão codificados e nenhuma intervenção será realizada, no entanto, caso sinais de complicações sejam identificados você notificará a equipe da unidade para que providências sejam tomadas de acordo com o protocolo institucional.

✓ Estudo piloto indicou que se dispende aproximadamente cinco minutos para preencher **o Formulário de Informações** e de três a quatro minutos para o **Formulário de Coleta de Dados** de avaliação dos primeiros CIP, mas que a medida que o avaliador se tornava mais familiarizado com os formulários este tempo diminuiu. O **Formulário de triagem do serviço/unidade** será preenchido no decorrer do dia da coleta com o intuito de prevenir a duplicação de dados.

✓ Todos os formulários do estudo estarão disponíveis para preenchimento on-line.

✓ Um *link* de pesquisa será enviado por e-mail para você preencher os formulários eletronicamente.

✓ Se você optar por preencher os formulários em papel, poderá inserir os dados por meio do *link* de pesquisa.

✓ Como alternativa você pode digitalizar e enviar por e-mail os formulários preenchidos para vascularstudybrazil@gmail.com.

Auditoria dos CIP

Espera-se que todos os CIP inseridos sejam auditados no dia do estudo de prevalência. Para a obtenção das informações que se caracterizam como uma auditoria, você poderá dirigir-se pessoalmente ou contatar por telefone o local de coleta dos dados para a obtenção das informações relacionadas ao CIP, assegurando que nenhuma informação pessoal, da história de saúde do paciente, da unidade e da instituição sejam coletadas.

Benefícios esperados

Espera-se que este estudo forneça informações abrangentes sobre padrões de cuidados com CIP em diferentes países. Este estudo terá importância internacional na documentação da prevalência do uso de CIP e suas complicações, como flebite, na América Latina. As informações obtidas serão de extrema relevância para o direcionamento de políticas e orçamentos no setor de saúde e fornecerão aos profissionais da área, gestores e empresas evidências que poderão ser traduzidas em ações de melhoria da prática. A natureza colaborativa do estudo também ajudará na construção de oportunidades de redes de trabalho e capacidade de pesquisa entre profissionais de saúde em diversos ambientes, o que facilitará o desenvolvimento benéfico de novas oportunidades de pesquisa no futuro. Como a auditoria relativa ao CIP será adicional à de rotina é possível que o avaliador possa identificar sinais precoces de complicações com CIP, como flebite ou infiltração. Nesse caso, o profissional notificará a equipe local. Portanto, pode haver um possível benefício para o paciente com CIP. Os possíveis benefícios para a instituição são:

• Oportunidades de *benchmarking* com outras organizações na sua região ou país, pois receberão os relatórios de resultados obtidos em sua instituição.
• Potencial para identificar oportunidades de melhoria da qualidade em sua organização, como uso de consumíveis (curativos, CIP, etc.), número de cateteres redundantes, etc.
• Potencial para o desenvolvimento de programas de educação direcionados a abordar áreas estratégicas, como a não conformidade com as diretrizes recomendadas, técnicas de curativos, etc.
• Potencial para publicação de descobertas específicas do local.

Riscos da Pesquisa

Não há riscos físicos previsíveis e nenhuma intervenção será implementada aos pacientes. As informações obtidas na auditoria são relativas às práticas usuais de uso de CIP. Riscos relacionados à quebra do sigilo quanto à identidade e informações do paciente serão observados e todas as medidas para a sua prevenção serão adotadas. A pesquisa implica em adicional trabalho para você, sendo esperado que o trabalho realizado seja retribuído a partir do retorno à instituição das informações coletadas com o adequado tratamento estatístico.

Confidencialidade

Cada organização receberá um código de identificação único. Nenhum detalhe sobre a identificação da instituição, do profissional que coletará os dados, das unidades ou de pacientes ou condições de saúde será revelado. Nenhum dado poderá ser rastreado levando à identificação do paciente. O compromisso com a privacidade e a confidencialidade dos dados utilizados será preservado integralmente. Não serão utilizadas informações em prejuízo das pessoas e ou da comunidade, inclusive em termos de autoestima, prestígio e ou econômico-financeiro. Os dados obtidos serão usados exclusivamente para finalidade prevista no protocolo do estudo.

Armazenamento dos dados

Será utilizado um processo rigoroso para garantir que toda informação das organizações participantes do estudo sejam confidenciais. Detalhes sobre CIP serão armazenados através de um aplicativo informatizado denominado REDCap, uma base de dados segura hospedada pela Universidade Griffith na Austrália e Universidade Federal de São Paulo. As instituições participantes que não optarem por uso da base de dados poderão registrar suas respostas através de formulários de coleta de dados em papel ou encaminhar por e-mail aos pesquisadores responsáveis no Brasil. Os formulários preenchidos serão arquivados e armazenados sob a guarda do pesquisador principal, bem como, os dados coletados por meio eletrônico e armazenados em computadores seguros alocados na Universidade de Griffith, Escola de Enfermagem e Obstetrícia, Brisbane, Austrália, e na Escola Paulista de Enfermagem da Unifesp, cujo acesso será igualmente compartilhado para a realização do estudo. As informações serão mantidas por período máximo de sete anos, de acordo entre as partes e segundo políticas locais.

Resultados

Os resultados desse estudo de prevalência serão publicados em periódicos científicos e apresentados em conferências nacionais e internacionais. Nenhum dado identificando qualquer instituição participante será divulgado. Não compartilharemos seus dados com outras instituições. Vamos publicar os resultados por país, não por hospital e os resultados individuais do hospital permanecerão confidenciais. Qualquer informação que você coletar permanece como propriedade de seu hospital.

Mérito Ético

A aprovação ética desta pesquisa foi obtida junto ao Comitê de Ética em Pesquisa da Universidade Griffith da Austrália (número de referência: 2018/292) e da Universidade Federal de São Paulo (Unifesp) sob o número 2.942.443, sendo submetido à Comissão Nacional de Ética em Pesquisa (CONEP, Brasil). Em casos de dúvidas entre em contato com o Comitê de Ética em Pesquisa (CEP) da Unifesp, Rua Prof. Francisco de Castro, no 55, CEP- 04020-050. E-mail: CEP@unifesp.edu.br, tel.: 5571-1062 ou com a Comissão Nacional de Ética Em Pesquisa – Conep SRTV 701, Via W 5 Norte, lote D - Edifício PO 700, 3o andar – Asa Norte CEP: 70719- 040, Brasília-DF, telefone (61) 3315-5877.

Nome do Pesquisador: Mavilde da Luz Gonçalves Pedreira - RG: 13335274-2
