# Supplementary material for: Peripheral vascular catheter use in Latin America (the vascular study): A multinational cross-sectional study
Source: Front Med (Lausanne). 2023 Jan 4;9:1039232. doi: 10.3389/fmed.2022.1039232 (PMC9846050; doi:10.3389/fmed.2022.1039232)
Supplement: Supplementary file 1 [file Data_Sheet_1.zip › Supplementary File 3.PDF]

Vascular AccesS Catheter Use in Latin AmeRica (VASCULAR study) Authorship Agreement Form

The VASCULAR study is a large study that reports / reports the prevalence, characteristics and management of peripheral venous catheters in Latin America. The results of the study will generate significant interest and will encourage organizations to use the information to inform the practice within their institutions.

The benefit of the study is that all information will remain the property of the hospital. This information can be used to compare with other hospitals locally, nationally or internationally. After the main study has been completed and the results of the research have been published in a peer-reviewed journal (or indexed journal) (all contributors / collaborators will be recognized in the publication), there will be an opportunity for individual hospitals or in groups publish your own data, encouraging us to this collaboration.

A condition to be part of this study is that no publication be sent to any journal or conference / congress until the results of the main study have been written and published in a peer-reviewed journal (or indexed magazine).

We also encourage you to collaborate with us, we can offer assistance / help in the analysis of the data and preparation of the manuscript. If you decide to publish your local data independently we ask you to recognize the VASCULAR team in any publication.

I, _________________________________ hereby become aware of and accept the terms of this author agreement and will acknowledge the VASCULAR team afterwards in any publication of the data.

Signature ______________________________ Date:__ /__ /____

Recognition to be specified in the publication:

"We would like to thank the study team Vascular Access Catheter use in Latin America (VASCULAR study) for the original idea and study design (Rachel Walker, Marie Cooke, Gillian Ray-Barruel, and Claire Rickard) ".

Estudio VASCULAR

Formulario de Acuerdo de Autoría

El estudio VASCULAR es un gran estudio que recolecta información acerca de la prevalencia, las características y el manejo de los catéteres venosos periféricos en América Latina. Los resultados del estudio generarán un gran interés y alentarán a las organizaciones a utilizar la información para dar a conocer la práctica dentro de sus instituciones

El beneficio del estudio es que toda la información seguirá siendo propiedad del hospital. Esta información puede usarse para compararla con otros hospitales a nivel local, nacional o internacional. Después de que el estudio principal haya sido completado y los resultados de la investigación hayan sido publicados en una revista revisada por pares (o revista indexada) (todos los participantes / colaboradores serán reconocidos en la publicación), habrá una oportunidad para hospitales de publicar sus propios datos cuando sea oportuno.

Una condición para ser parte de este estudio es que no se envíe ninguna publicación a ninguna revista o conferencia/congreso hasta que los resultados del estudio principal hayan sido escritos y publicados en una revista revisada por pares. (o revista indexada)

También le recomendamos que colabore con nosotros, podemos ofrecer asistencia/ayuda en el análisis de los datos y la preparación del manuscrito. Si decide publicar sus datos locales de forma independiente, le pedimos que reconozca al equipo de VASCULAR en cualquier publicación.

Yo, ______________________________, tomo conocimiento y acepto los términos de este acuerdo de autor y reconoceré al equipo de VASCULAR posteriormente en cualquier publicación de los datos.

____________________________ Fecha:__ /__ /____

Firma

Reconocimiento a ser especificado en la publicación:

"Nos gustaría agradecer al equipo de estudio Vascular Access Catheter en América Latina (estudio VASCULAR) por la idea original y el diseño del estudio (Rachel Walker, Marie Cooke y Claire Rickard)".

Prezado Participante.

Primeiramente agradecemos a anuência e parceria para o desenvolvimento do Estudo VASCULAR, pesquisa transversal e multinacional, que tem como finalidade verificar a prevalência de uso, modos de avaliação e manuseio de cateteres intravenosos periféricos (CIP) em hospitais da América Latina.

Os dados obtidos na pesquisa serão usados exclusivamente para as finalidades previstas no protocolo do estudo, resultando em apresentação em eventos e publicação de artigos científicos em periódicos revisados por pares, com posterior retorno de relatório de resultados a cada instituição, individualmente. Os dados serão mantidos armazenados nas universidades brasileira e australiana, sendo devolvidos às instituições participantes, após a divulgação dos estudos multinacionais e nacionais.

Após a publicação dos estudos principais, em periódicos revisados por pares (revistas indexadas) haverá devolutiva dos dados para a sua instituição, sendo possível analisar e publicar seus próprios dados, ou realizar apresentações em eventos ou fóruns científicos e técnicos.

Após a divulgação/publicação dos resultados dos estudos principais, multinacionais e nacionais, solicitamos que seja reconhecida, na forma de agradecimento, a equipe que idealizou o estudo, conforme modelo apresentado abaixo*.

Encorajamos a todos os participantes a colaborarem na disseminação de resultados, a fim de que possamos contribuir com o avanço do conhecimento na área. Os grupos de pesquisa podem oferecer assistência para a análise dos dados e preparação de manuscritos e relatórios.

Eu, ____, do Hospital/instituição ____________________________________, participante do estudo VASCULAR, entendi e concordo com os termos deste acordo de autoria, para aguardar o prazo de publicações principais para prosseguir com adicionais divulgações, bem como, reconhecer em agradecimento a equipe do estudo VASCULAR.

Assinatura:____Data de assinatura:____ /____ /____

*Reconhecimento a ser especificado na publicação:

"We would like to thank the Vascular Access Catheter study team in Latin America (VASCULAR study) for the original idea and design of the study (Rachel Walker, Maria Isabel Castillo, Mavilde Pedreira, Marie Cooke, Maria Paula Pires, Gillian-Ray Barruel, Claire Rickard)".

"Gostaríamos de agradecer à equipe de estudo do Vascular Access Catheter Use in Latin America (estudo VASCULAR) pela ideia original e desenho do estudo (Rachel Walker, Maria Isabel Castillo, Mavilde Pedreira, Marie Cooke, Maria Paula Pires, Gillian-Ray Barruel , Claire Rickard)".
