# Supplementary material for: Peripheral vascular catheter use in Latin America (the vascular study): A multinational cross-sectional study
Source: Front Med (Lausanne). 2023 Jan 4;9:1039232. doi: 10.3389/fmed.2022.1039232 (PMC9846050; doi:10.3389/fmed.2022.1039232)
Supplement: Supplementary file 1 [file Data_Sheet_1.zip › Supplementary File 4.DOCX]

**SUPPLEMENTARY FILE 4 – SITE INFORMATION FORM**

Form 1 – Site information form in English version


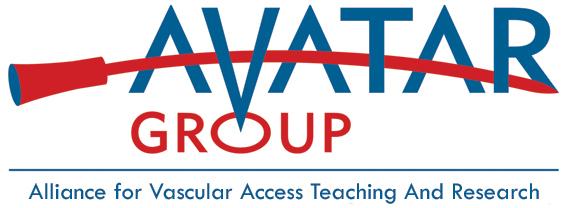


| **Country** |  |
| --- | --- |
| **Region/State/Province** |  |
| **Hospital/Site** |  |
| **Number of hospital beds** |  |
| **Date of review** |  |

**
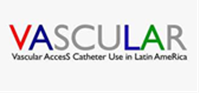
**

**VASCULAR PIVC Study Site Information Form**

***Please complete the following details about the use of peripheral intravenous catheters (PIVC) at this site.***

Q 1.1 Role of person completing this form

| 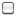 | Intravenous Nurse Specialist | 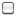 | Nurse Researcher |
| --- | --- | --- | --- |
| 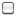 | Intravenous Medical Specialist | 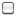 | Medical Researcher |
| 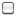 | Intravenous Technician | 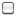 | Research Assistant |
| 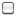 | Clinical Nurse Specialist/Consultant | 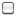 | Staff Nurse |
| 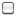 | Infection Control Nurse | 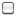 | Administrative Staff |
| 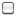 | Nurse Educator | 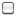 | Other ____________________ |

**PIVC GUIDELINES**

| Q 2.1 Does your hospital/site have PIVC insertion and maintenance guidelines for staff? | 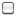 Yes | 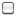 No |
| --- | --- | --- |

**If yes, please complete the following regarding your hospital’s PIVC guidelines.**

| Q 2.2 Do these guidelines state how often to change the PIVC? | 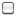 Yes | 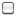 No |
| --- | --- | --- |

Q 2.3 What is the guideline for frequency of replacing the PIVC?

| 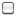 | 24–48 hours |
| --- | --- |
| 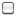 | 48–72 hours |
| 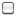 | 72–96 hours |
| 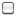 | >96 hours |
| 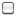 | Clinically indicated |
| 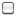 | Other _______________________ |

| Q 2.4 Do these guidelines state how often to change the PIVC dressing? | 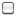Yes | 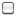No |
| --- | --- | --- |

| 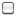 | 24 hours and prn |
| --- | --- |
| 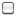 | 48 hours and prn |
| 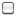 | 72hours and prn |
| 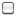 | 96 hours and prn |
| 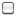 | > 96 hours and prn |
| 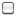 | PRN only |

Q 2.5 What is the recommended frequency of PIVC dressing change?

| Q 2.6 Do these guidelines state how often to change the PIVC administration set? | 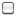Yes | 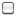No |
| --- | --- | --- |

Q 2.7 What is the recommended frequency of administration set change for crystalloid fluids (saline or dextrose solutions)?

| 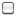 | 24–48 hours |
| --- | --- |
| 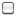 | 48–72 hours |
| 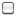 | 72–96 hours |
| 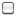 | 96 hours–7 days |
| 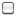 | > 7 days |
| 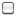 | Other _______________________ |

| Q 2.8 Does your hospital have guidelines for PIVC site assessment? | 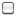Yes | 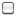No |
| --- | --- | --- |

Q 2.9 If yes, what is the recommended frequency of PIVC site assessment? (check all that apply)

| 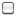 | Every 4 hours |
| --- | --- |
| 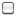 | Every 8 hours or once per 8 hours shift |
| 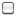 | Every 12 hours or once per 12 hours shift |
| 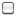 | Once daily |
| 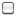 | Second daily |
| 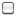 | Every time the catheter is used |
| 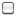 | Other ____________________________ |

**PHLEBITIS SCORING TOOLS**

| Q 3.1 Is a phlebitis scale/scoring tool in use at this hospital/site? | 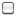Yes | 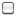No | 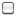Unknown |
| --- | --- | --- | --- |

Q 3.2 If yes, what is the name of the scale (if known)?

| 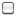 | Infusion Nurses Society (INS) scale |
| --- | --- |
| 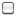 | Visual Infusion Phlebitis (VIP) score |
| 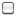 | Baxter scale |
| 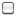 | PVC ASSESS scale |
| 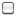 | Maddox scale |
| 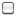 | Jackson scale |
| 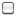 | Other _____________________________ |

**PIVC INSERTION**

| Q 4.1 Is a dedicated, single use, sterile PIVC insertion pack used for PIVC insertions at this hospital/site? | 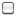Yes | 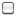No | 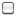Unknown |
| --- | --- | --- | --- |

Q 4.2 What is the recommended PIVC insertion technique at this site:

| 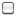 | Clean |
| --- | --- |
| 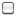 | Aseptic non-touch technique |
| 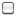 | Strictly aseptic technique |
| 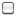 | Unknown |

| Q 4.3 Is hand hygiene regularly audited? | 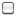Yes | 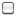No | 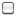Unknown |
| --- | --- | --- | --- |
| Q 4. Is blood return compulsory before the PIVC is used for intravenous therapy? | 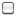Yes | 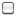No | 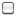Unknown |

Q 4.4 Who inserts the PIVCs at this hospital/site? (check all that apply)

| 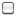 | Specialist IV team |
| --- | --- |
| 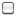 | Nurses |
| 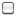 | IV Technicians |
| 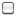 | Doctors |
| 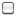 | Physicians’ Assistants |
| 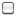 | Other ____________________ |

| Q 4.5 Is PIVC insertion training provided for nurses? | 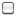Yes | 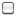No | 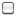Unknown |
| --- | --- | --- | --- |

Q 4.6 If training for IV insertion is provided for nurses, how many hours of theoretical training?

| 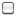 | 4 hours |
| --- | --- |
| 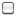 | 8 hours |
| 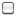 | 2 days |
| 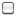 | Other ________________________ |

| Q 4.7 How many successful attempts are required for competency for nurses (if known | __________attempts |
| --- | --- |

| Q 4.8 Is PIVC insertion training provided for doctors? | 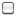Yes | 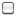No | 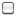Unknown |
| --- | --- | --- | --- |

Q 4.9 If training for IV insertion is provided for doctors, how many hours of theoretical training?

| 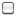 | 4 hours |
| --- | --- |
| 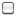 | 8 hours |
| 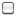 | 2 days |
| 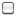 | Other ________________________ |

| Q 4.10 How many successful attempts are required for competency for doctors (if known)? | __________attempts |
| --- | --- |

| Q 4.11 Is it mandatory that PIVC inserters are assessed as competent at regular intervals? | 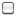Yes | 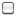No | 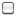Unknown |
| --- | --- | --- | --- |

Q 4.12 If yes, what is the frequency of competency re-assessment?

| 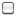 | 1 year |
| --- | --- |
| 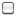 | 2 years |
| 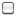 | 3 years |
| 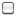 | Other ________________________ |

| Q 4.13 Is there a dedicated specialist IV team for peripheral IV cannulation at this site? | 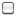Yes | No | Unknown |
| --- | --- | --- | --- |

| Q 4.14 If yes, do you agree to be contacted for further information about the make-up of this team? | Yes | No |
| --- | --- | --- |

| Q 4.15 Are other vascular access specialists available to support difficult PIVC insertion? | Yes | No | Unknown |
| --- | --- | --- | --- |

Q 4.16 If yes, what is their designated role?

|  | Clinical Nurse Specialist/Consultant |  |
| --- | --- | --- |
|  | Anesthetist/Intensivist |  |
|  | Phlebotomist |  |
|  | Other |  |

Q 4.17 Which PIVC insertion method is most commonly used in this hospital/site?

|  | Traditional (landmark, palpation) |
| --- | --- |
|  | Technology (guided insertion) |
|  | Unknown |

| Q 4.18 If guided PIVC insertion technology is used, do you agree to be contacted for further information? | Yes | No |
| --- | --- | --- |

**CLEANING SOLUTIONS**

Q 5.1 Which cleaning solutions are used for PIVC insertion and dressing changes at this hospital?

(check all that apply)

| **Solution** | **Insertion** | **Dressing changes** |
| --- | --- | --- |
| Chlorhexidine 2% in 70% alcohol |  |  |
| Chlorhexidine 1% in 70% alcohol |  |  |
| Chlorhexidine 0.5% in 70% alcohol |  |  |
| Chlorhexidine without alcohol |  |  |
| Povidone-iodine in alcohol |  |  |
| Povidone-iodine without alcohol |  |  |
| 70–75% alcohol |  |  |
| 0.9% sodium chloride |  |  |
| Other (specify) __________________________ |  |  |

**PIVC DRESSINGS**

Q 6.1 Which PIVC dressings are used at this hospital? (check all that apply)

|  | | *Brand (if known)* |
| --- | --- | --- |
|  | Borderless transparent polyurethane dressing |  |
|  | Window transparent polyurethane dressing |  |
|  | Sterile gauze and tape dressing |  |
|  | Chlorhexidine-impregnated dressing |  |
|  | Other (specify) |  |

**PIVC BRANDS AND PRODUCTS**

Q 7.1 Which PIVC brands and products are used at this hospital? (check all that apply)

| **BRANDS** | | **PRODUCTS** | | | |
| --- | --- | --- | --- | --- | --- |
|  | Becton Dickinson (BD) | Adsyte™ Pro  Neoflon™  Saf-T-Intima™ | AngioCath  Nexiva™  Venflon™ | Insyte™ Autoguard  Pegasus  Other _____________ | |
|  | B Braun | Introcan  Vasofix® Braunüle®  Other _________________ | Introcan® Certo  Vasofix® Certo | Introcan Safety®  Vasofix® Safety | |
|  | Exel | Exel IV catheter | Other _________________ | | |
|  | Gambro | Supercath™ | Other _________________ | | |
|  | Lars Medicare | Other _________________ |  | |  |
|  | MediPlus | Pluscan  Other _________________ | Pluscan Alpha | | Plusneo |
|  | MedSource | MedSource IV Safety | Other _________________ | | |
|  | Polymed | Other _________________ |  |  | |
|  | Smiths Medical | Acuvance®  Jelco®  ProtectIV® | AdvantIV®  Jelco ViaValve®  ProtectIV® PLUS | Cathlon®  Optiva®  Other ____________ | |
|  | Terumo | Surflo® | Surflash® | Other ____________ | |
|  | Tangent Medical | NovaCath™ | Other ________________ | | |
|  | Vygon | BioValve® safe | Other________________ | | |
|  | Unknown |  |  | |  |

Please enter your completed data on the on-line survey link provided via email, or contact us for postage details or scan and email completed form to [m.arneil@griffith.edu.au](mailto:m.arneil@griffith.edu.au)

Thank you!

Form 2 – Site information form in Portuguese version

| **País** |  |
| --- | --- |
| **Estado/Cidade** |  |
| **Hospital** |  |
| **Número de leitos do hospital** |  |
| **Data da coleta** |  |

**Estudo** **VASCULAR**

**Formulário de Informações da Instituição Participante**

***Por favor, complete as informações detalhadamente sobre a utilização de cateteres intravenosos periféricos (CIP) nesta instituição.***

Q 1.1 Cargo do profissional que preencherá este formulário:

|  | Enfermeiro especialista IV |  | Médico pesquisador |
| --- | --- | --- | --- |
|  | Médico da equipe IV |  | Assistente de pesquisa |
|  | Técnico de enfermagem |  | Enfermeiro Assistencial |
|  | Enfermeiro clínico especialista |  | Equipe de administração |
|  | Enfermeiro da CCIH |  | Enfermeiro Gestor |
|  | Enfermeiro Educador/ Docente |  | Médico/ Residente |
|  | Enfermeiro Pesquisador |  | Outro ____________________ |

**RECOMENDAÇÕES PARA O USO DE CIP**

| Q 2.1 O seu hospital possui protocolos para inserção e manutenção de cateteres intravenosos periféricos (CIP)? | Sim | Não |
| --- | --- | --- |

**Se sim, por favor, complete as seguintes questões acerca das recomendações do seu hospital:**

| Q 2.2 Estes protocolos determinam a frequência para a troca dos CIP? | Sim | Não |
| --- | --- | --- |

Q 2.3 Qual é a recomendação para a troca dos CIP?

|  | **Adultos:** |  | **Crianças:** |
| --- | --- | --- | --- |
|  | 24–48 horas |  | 24–48 horas |
|  | 48–72 horas |  | 48–72 horas |
|  | 72–96 horas |  | 72–96 horas |
|  | >96 horas |  | >96 horas |
|  | Quando clinicamente indicado |  | Quando clinicamente indicado |
|  | Outro _______________________ |  | Outro _______________________ |

| Q 2.4 Os protocolos determinam a frequência para troca do curativo/cobertura do CIP? | Sim | Não |
| --- | --- | --- |

|  | 24 horas e se necessário |
| --- | --- |
|  | 48 horas e se necessário |
|  | 72 horas e se necessário |
|  | 96 horas e se necessário |
|  | > 96 horas e se necessário |
|  | Somente se necessário |

Q 2.5 Qual é a recomendação mais utilizada para a troca do curativos do CIP?

| Q 2.6 Os protocolos determinam a frequência para troca dos sistemas de infusão de CIP? | Sim | Não |
| --- | --- | --- |

Q 2.7 Qual é a frequência recomendada para troca dos sistemas de infusão de fluidos cristalóides (soluções com soro fisiológico ou soro glicosado)?

|  | 24–48 horas |
| --- | --- |
|  | 48–72 horas |
|  | 72–96 horas |
|  | 96 horas –7 dias |
|  | > 7 dias |
|  | Outro ______________________ |

| Q 2.8 O seu hospital tem protocolos para avaliação do sítio de inserção do CIP? | Sim | Não |
| --- | --- | --- |

Q 2.9 Se sim, qual é a frequência recomendada para avaliação do sítio de inserção dos CIP? (assinalar as opções aplicáveis)

|  | A cada 4 horas |
| --- | --- |
|  | A cada 6 horas ou uma vez por plantão (de 6 horas) |
|  | A cada 8 horas ou uma vez por plantão (de 8 horas) |
|  | A cada 12 horas ou uma vez por plantão (de 12 horas) |
|  | Uma vez por dia (24 horas) |
|  | A cada dois dias (48 horas) |
|  | Sempre que o cateter é utilizado |
|  | Outro ____________________________ |

**INSTRUMENTOS PARA AVALIAÇÃO DE FLEBITE**

| Q 3.1 No seu hospital, é utilizada uma escala/score para avaliação de flebite? | Sim | Não | Não se sabe |
| --- | --- | --- | --- |

Q 3.2 Caso sim, qual escala?

|  | Escala da Infusion Nurses Society (INS) |
| --- | --- |
|  | Escala da Visual Infusion Phlebitis (VIP) |
|  | Escala Baxter |
|  | Escala PVC ASSESS |
|  | Escala Maddox |
|  | Escala Jackson |
|  | Outra _____________________________ |

**INSERÇÃO CIP**

| Q 4.1 É utilizado um kit de uso único para inserção de CIP no hospital? | Sim | Não | Não se sabe |
| --- | --- | --- | --- |

Q 4.2 Qual é a técnica de inserção recomendada?

|  | Limpa |  |
| --- | --- | --- |
|  | Técnica asséptica “no-touch” (sem toque no local da inserção) | |
|  | Técnica estritamente asséptica (com uso de luva estéril) |  |
|  | Desconhecido |  |

| Q 4.3 A higienização das mãos é frequentemente auditada? | Sim | Não | Não se sabe |
| --- | --- | --- | --- |
| Q 4.4 É obrigatório o retorno de sangue antes do uso do CIP para a terapia intravenosa? | Sim | Não | Não se sabe |

Q 4.5 Quem insere CIP no seu hospital? (assinale todas as opções possíveis)

|  | Equipe de terapia IV |
| --- | --- |
|  | Enfermeiros |
|  | Técnico de Enfermagem/ Auxiliar de Enfermagem/ Técnicos de laboratório |
|  | Médico |
|  | Médicos residentes |
|  | Outro ____________________ |

| Q 4.6 Os enfermeiros do seu hospital recebem treinamento de inserção de CIP? | Sim | Não | Não se sabe |
| --- | --- | --- | --- |

Q 4.7 Caso os enfermeiros recebam o treinamento, quantas horas de atividade teórica?

|  | 4 horas |
| --- | --- |
|  | 8 horas |
|  | 2 dias |
|  | Outro ________________________ |

| Q 4.8 Quantas tentativas com sucesso são necessárias para atestar a competência dos enfermeiros para a punção (se conhecido)? ______ tentativas Não se sabe |
| --- |

| Q 4.9 Os médicos do seu hospital recebem treinamento de inserção de CIP? | Sim | Não | Não se sabe |
| --- | --- | --- | --- |

Q 4.10 Caso os médicos recebam o treinamento, quantas horas de atividade teórica?

|  | 4 horas |
| --- | --- |
|  | 8 horas |
|  | 2 dias |
|  | Outro ________________________ |

| Q 4.11 Quantas tentativas com sucesso são necessárias para atestar a competência dos médicos para a punção (se conhecido)? ______ tentativas Não se sabe |
| --- |

| Q 4.12 É obrigatória a avaliação de competência dos profissionais que inserem CIP em intervalos regulares? | Sim | Não | Nao se sabe |
| --- | --- | --- | --- |

Q 4.13 Caso sim, qual é a frequência de reavaliação de competências?

|  | 1 ano |
| --- | --- |
|  | 2 anos |
|  | 3 anos |
|  | Outro ________________________ |

| Q 4.14 No seu hospital, existe um time de especialistas em terapia IV para a inserção de CIP? | Sim | Não | Não se sabe |
| --- | --- | --- | --- |
| Q 4.15 Caso sim, você concorda em ser contatado futuramente para informações sobre a composição desse time? | Sim | Não |  |
| Q 4.16 Existem outros especialistas em acesso vascular disponíveis para auxiliarem em casos difíceis nas inserções de CIP? | Sim | Não | Não se sabe |

Q 4.17 Caso sim, qual é a sua função?

|  | Enfermeiro clínico especialista/Consultor |  |
| --- | --- | --- |
|  | Anestesista/Intensivista |  |
|  | Médico/Flebotomista |  |
|  | Outro |  |

Q 4.18 Qual o método de inserção de CIP mais utilizado no seu hospital?

|  | Tradicional (referência anatômica, palpação) |
| --- | --- |
|  | Inserção guiada por tecnologia |
|  | Desconhecido |

| Q 4.19 Se for utilizada tecnologia de inserção guiada, concorda em ser contatado para maiores informações? | Sim | Não |
| --- | --- | --- |

**SOLUÇÕES ANTISSÉPTICAS**

Q 5.1 No seu hospital, quais as soluções antissépticas são utilizadas para inserção e troca de curativo de CIP? (assinale todas as opções possíveis)

| **Solução** | **Inserção** | **Troca de curativo** |
| --- | --- | --- |
| Clorexidina alcoólica a 2% |  |  |
| Clorexidina alcoólica a 1% |  |  |
| Clorexidina alcoólica a 0.5% |  |  |
| Clorexidina aquosa (sem álcool) |  |  |
| PVPI alcoólico |  |  |
| PVPI sem álcool |  |  |
| Álcool a 70–75% |  |  |
| Soro Fisiológico |  |  |
| Outro (especificar) __________________________ |  |  |
| Outro (especificar) __________________________ |  |  |

**TROCA DE CURATIVOS/COBERTURAS do CIP**

Q 6.1 Quais os curativos utilizados para os CIP no seu hospital? (assinale as opções possíveis)

|  | | *Marca (se conhecer)* |
| --- | --- | --- |
|  | Película transparente em poliuretano sem borda | ___________________________________________ |
|  | Película transparente em poliuretano com borda | ___________________________________________ |
|  | Gaze estéril com fita adesiva | ___________________________________________ |
|  | Fita adesiva | ___________________________________________ |
|  | Curativo impregnado com clorexidina | ___________________________________________ |
|  | Outro (especificar) | ___________________________________________ |

**MARCAS E PRODUTOS CIP**

Q 7.1 Quais as marcas dos CIP e produtos utilizados no seu hospital? (assinalar as opções aplicáveis)

| Becton Dickinson (BD) | | Adsyte™ Pro  Neoflon™  Saf-T-Intima™ | AngioCath  Nexiva™  Venflon™ | | | Insyte™ Autoguard  Pegasus  Outro _____________ | | |
| --- | --- | --- | --- | --- | --- | --- | --- | --- |
|  | B Braun | Introcan  Vasofix® Braunüle®  Outro _________________ | | | Introcan® Certo  Vasofix® Certo | | Introcan Safety®  Vasofix® Safety | |
|  | Exel | Exel IV catheter | | | Outro _________________ | |  | |
|  | Gambro | Supercath™ | | | Outro _________________ | | | |
|  | Lars Medicare | Outro _________________ | | |  | | | |
|  | MediPlus | Pluscan  Outro _________________ | | | Pluscan Alpha | | | Plusneo |
|  | MedSource | MedSource IV Safety | | | Outro _________________ | | |  |
|  | Polymed | Outro _________________ | | |  | | | |
|  | Smiths Medical | Acuvance®  Jelco®  ProtectIV® | | | AdvantIV®  Jelco ViaValve®  ProtectIV® PLUS | | Cathlon®  Optiva®  Outro ____________ | |
|  | Terumo | Surflo® | | | Surflash® | | Outro ____________ | |
|  | Tangent Medical | NovaCath™ | | | Outro ________________ | |  | |
|  | Vygon | BioValve® safe | | | Outro________________ | | | |
|  | Outro____________________ | Outro __________________ | | Outro ____________________________ | | | | |
|  | Outro____________________ | Outro___________________ | | Outro __________________ | | | | |
|  | Outro___________________ | Outro___________________ | | Outro __________________ | | | | |
|  | Outro___________________ | Outro___________________ | | Outro __________________ | | | | |
|  | Desconhecido |  | | |  | | |  |

Por favor, introduza os dados no link da pesquisa fornecido via e-mail ou contate-nos para qualquer detalhe. O nosso e-mail para contato é vascularstudybrazil@gmail.com

Obrigado!

Form 3 – Site information form in Spanish

| **País** |  |
| --- | --- |
| **Ciudad** |  |
| **Hospital/Sitio** |  |
| **Número de camas hospitalarias** |  |
| **Fecha de la evaluación** |  |

Estudio VASCULAR

**Formulario de Información del sitio del Estudio**

***Por favor completar los siguientes detalles acerca del uso de catéteres venosos periféricos cortos (CVPC) en este sitio.***

Q 1.1 Rol de la persona que complete el formulario:

| 🞏 | Enfermera del equipo terapia IV | 🞏 | Investigador médico |
| --- | --- | --- | --- |
| 🞏 | Médico Equipo terapia IV | 🞏 | Asistente de investigación |
| 🞏 | Técnico (diagnóstico/hemoterapia/anestesia) | 🞏 | Enfermero asistencial/ Lic enfermería |
| 🞏 | Especialista en enfermería clínica/consultora | 🞏 | Personal administrativo |
| 🞏 | Enfermera de control de infecciones | 🞏 | Enfermero de gerencia/gestión |
| 🞏 | Enfermera educadora/docente | 🞏 | Médico/Residente |
| 🞏 | Investigador de enfermería | 🞏 | Otros |

## GUÍAS O PROTOCOLOS SOBRE CVPC

| Q 2.1 ¿Su hospital tiene guías o protocolos para la colocación/inserción y mantenimiento de catéter venoso periférico corto (CVPC)? | Sí | No |
| --- | --- | --- |
| **En caso afirmativo, por favor complete lo siguiente con respecto a las pautas CVPC de su hospital** | | |
| Q 2.2 ¿Estás guías indican con qué frecuencia cambiar el catéter venoso periférico corto (CVPC)? | Sí | No |

Q 2.3 ¿Cuál es la frecuencia de cambio CVPC, que dice el protocolo?

|  | **Adultos:** |  | **Niños:** |
| --- | --- | --- | --- |
|  | 24–48 horas |  | 24–48 horas |
|  | 48–72 horas |  | 48–72 horas |
|  | 72–96 horas |  | 72–96 horas |
|  | >96 horas |  | >96 horas |
|  | Indicación clínica |  | Indicación clínica |
|  | Otros _ |  | Otros _ |

| Q 2.4 ¿Los protocolos/guías establecen la frecuencia de cambio de la  curación/apósito del CVPC? | Sí | No |
| --- | --- | --- |

Q 2.5 ¿Cuál es la frecuencia recomendada de cambio de la curación/apósito del CVPC?

|  | 24 horas y prn |
| --- | --- |
|  | 48 horas y prn |
|  | 72 horas y prn |
|  | 96 horas y prn |
|  | > 96 horas y prn |
|  | Cuando es necesario/*pro re nata* (PRN: Criterio del personal de enfermería) |

| Q 2.6 ¿Los protocolos establecen con qué frecuencia cambiar la  tubuladura/equipo de administración de CVPC? | Sí | No |
| --- | --- | --- |

Q 2.7 ¿Cuál es la frecuencia recomendada de cambio de tubuladuras/equipo de administración de fluidos cristaloides (ex: solución salina o dextrosa)?

|  | 24–48 horas |
| --- | --- |
|  | 48–72 horas |
|  | 72–96 horas |
|  | 96 horas –7 dias |
|  | > 7 dias |
|  | Otros |

| Q 2.8 ¿Su hospital tiene guías/protocolos para la valoración del sitio de  inserción del CVPC? | Sí | No |
| --- | --- | --- |

Q 2.9 Si es sí, ¿cuál es la frecuencia recomendada para la valoración del sitio de inserción del CVPC? (marcar todo lo que corresponda)

Cada hora

|  | Cada 4 horas |
| --- | --- |
|  | Cada 6 horas o una vez por turno de 6 horas |
|  | Cada 8 horas o una vez por turno de 8 horas |
|  | Cada 12 horas o una vez por turno de 12 horas |
|  | Una vez al día (24 hs) |
|  | Cada dos dias (48 hs) |
|  | Cada vez que se utiliza el catéter |
|  | Otros _ |

| **ESCALA DE FLEBITIS**  Q 3.1 ¿Se utiliza alguna escala de flebitis en su hospital? | Sí | No | No sabe |
| --- | --- | --- | --- |

Q 3.2 Si es sí, ¿cuál es el nombre de la escala (si se conoce)?

|  | Escala de la Sociedad de Enfermería de Infusión (INS) |
| --- | --- |
|  | Escala Visual Infusión flebitis (VIP) |
|  | Escala de Baxter |
|  | Escala ASSESS PVC |
|  | Escala Maddox |
|  | Escala de Jackson |
|  | Otro _ |

## COLOCACIÓN/INSERCIÓN DEL CVPC

| Q 4.1 ¿Se dispone de un set/paquete de un solo uso, para la  inserción de CVPC en este sitio/hospital? | Sí | No | No se sabe |
| --- | --- | --- | --- |

Q 4.2 ¿Cuál es la técnica de inserción/colocación recomendada en este sitio/hospital?

|  | Limpia |
| --- | --- |
|  | Técnica aséptica (sin tocar) |
|  | Técnica aséptica estricta |
|  | No se sabe |

| Q 4.3 ¿Es auditada la higiene de las manos regularmente? | Sí | No | No se sabe |
| --- | --- | --- | --- |
| Q 4.4 ¿Es obligatorio el retorno de la sangre antes de usar el CVPC para la terapia intravenosa? | Sí | No | No se sabe |

Q 4.5 ¿Quién inserta los CVPCs en este hospital/sitio? (marcar todo lo que corresponda)

|  | Equipo IV |  |  |  |
| --- | --- | --- | --- | --- |
|  | Enfermeros/Licenciado en enfermeria | |  |  |
|  | Técnico (diagnóstico/hemoterapia/anestesia) | |  |  |
|  | Auxiliar de Enfermería |  |  |  |
|  | Paramédico |  |  |  |
|  | Médico/Residente |  |  |  |
|  | Estudiante de Enfermería |  |  |  |
|  | Estudiante de Medicina |  |  |  |
|  | Otros |  |  |  |
| Q 4.6 ¿Se entrena/forma a los enfermeros para la inserción de  CVPC? | | Sí | No | No se sabe |

Q 4.7 Si se entrena a los enfermeros en la inserción de CVPC, ¿Cuántas horas de formación teórica se imparten?

|  | 4 horas |
| --- | --- |
|  | 8 horas |
|  | 2 dias |

|  | Otro |
| --- | --- |

Q 4.8 ¿Cuantas CVPC exitosas son necesarias para acreditar la competencia? Inserciones (Enfermeras) No se sabe

| Q 4.9 ¿Se entrena/forma a los médicos en la inserción de CVPC? | Sí | No | No se sabe |
| --- | --- | --- | --- |

Q 4.10 Si se entrena a los médicos en la inserción de CVPC, ¿Cuántas horas de formación teórica se imparten?

|  | 4 horas |
| --- | --- |
|  | 8 horas |
|  | 2 dias |
|  | Otro |

Q 4.11 ¿Cuantas inserciones de CVPC exitosas son necesarias para acreditar la competencia? _ Inserciones (Médicos) No se sabe

| Q 4.12 ¿Es obligatoria la evaluación de la competencia a  intervalos regulares de los que colocan los CVPCs? | Sí | No | No se sabe |
| --- | --- | --- | --- |

Q 4.13 Si es sí, ¿cuál es la frecuencia de re-evaluación de la competencia?

|  | 1 año |  |  |  |
| --- | --- | --- | --- | --- |
|  | 2 años |  |  |  |
|  | 3 años |  |  |  |
|  | Otro |  |  |  |
| Q 4.14 ¿Su hospital tiene un equipo IV especialista dedicado para la colocación/inserción de CVPC? | | Sí | No | No se sabe |

| Q 4.15 Si es sí, ¿está de acuerdo en ser contactado para mayor información acerca de la composición/organización de este equipo? | Sí, e-mail: | No |
| --- | --- | --- |

| Q 4.16 ¿Hay otros especialistas en accesos vasculares disponibles  para soporte en las inserciones de CVPCs difíciles? | Sí | No | No se sabe |
| --- | --- | --- | --- |

Q 4.17 Si es sí, ¿cuál es el rol resignado? (marcar todo lo que corresponda)

|  | Enfermero Especialista Clínico / Consultor |
| --- | --- |
|  | Anestesista / Intensivista |
|  | Flebotomista |
|  | Otro _ |

Q 4.18 ¿Cuál es el método de inserción mas común usado en este sitio/hospital?

|  | Tradicional (palpación de un punto de referencia) |
| --- | --- |
|  | Colocación guiada por tecnología |
|  | No se sabe |

| Q 4.19 Si utiliza la colocación/inserción del CVPC guiada por tecnología,  ¿está de acuerdo en ser contactado para mayor información? | Sí | No |
| --- | --- | --- |

# SOLUCIONES ANTISEPTICAS

Q 5.1 ¿Cuáles son las soluciones en uso para la colocación de CVPC y cambios de apósitos en este hospital? (marcar todo lo que corresponda)

| **Soluciones** | **Inserción** | **Cambios de apósitos** |
| --- | --- | --- |
| Clorhexidina al 2% en alcohol al 70% |  |  |
| Clorhexidina al 1% en alcohol al 70% |  |  |
| Clorhexidina al 0.5% en alcohol al 70% |  |  |
| Clorhexidina acuosa (sin alcohol) |  |  |
| Yodopovidona/povidona yodada en alcohol |  |  |
| Yodopovidona/povidona yodada sin alcohol |  |  |
| Alcohol 70-75% |  |  |
| Cloruro de sodio al 0.9% |  |  |
| Otro (especificar) _ |  |  |
| Otro (especificar) _ |  |  |

# TIPOS DE APÓSITOS DE CVPC

Q 6.1 ¿Qué tipos de apósitos de CVPC se utilizan en este hospital? (marcar todo lo que corresponda)

|  |  | *Marca (si se conoce)* |
| --- | --- | --- |
|  | Apósito de poliuretano transparente sencillo |  |
|  | Apósito de poliuretano transparente con bordes |  |
|  | Gasa estéril y tela adhesiva  Vendaje de cinta estéril |  |
|  | Cintas adhesivas (no estéril) |  |
|  | Apósito impregnado con Clorhexidina |  |
|  | Otros (especificar) |  |

# MARCAS/PRODUCTOS DE CVPC

Q 7.1 ¿Cuáles Marcas/productos de catéter venoso periférico corto CVPC en uso en este hospital? (Nómbrelos)

| Becton Dickinson (BD) | | Adsyte™ Pro Neoflon™  Saf-T-Intima™ | AngioCath  Nexiva™ Venflon™ | | Insyte™ Autoguard  Pegasus  Otro |
| --- | --- | --- | --- | --- | --- |
|  | B Braun | Introcan Vasofix® Braunüle®  Otro |  | Introcan® Certo Vasofix® Certo | Introcan Safety® Vasofix® Safety |
|  | Exel | Exel IV catheter | | Otro  _ | |
|  | Gambro | Supercath™ | | Otro | |
|  | Lars Medicare | Otro | |  |  |
|  | MediPlus | Pluscan  Otro |  | Pluscan Alpha | Plusneo |
|  | MedSource | MedSource IV Safety |  | Otro | |
|  | Polymed | Otro | | | |
|  | Smiths Medical | Acuvance®  Jelco® ProtectIV® |  | AdvantIV®  Jelco ViaValve® ProtectIV® PLUS | Cathlon® Optiva®  Otro _ |
|  | Terumo | Surflo® |  | Surflash® | Otro _ |
|  | Tangent Medical | NovaCath™ |  | Otro _ |  |
|  | Vygon | BioValve® safe | | Otro | |
|  | Otro _ | Otro | | Otro _ | |
|  | Otro _ | Otro |  | Otro | |
|  | Otro | Otro |  | Otro | |
|  | Otro | Otro |  | Otro | |
|  | Desconocido |  | | | |

Por favor introduzca los datos completos en la página web de la encuesta por medio del vínculo enviado por mail, o contáctenos para los detalles de envío postal, o escaneo y envío por correo electrónico a [vascularstudybrazil@gmail.com](mailto:vascularstudybrazil@gmail.com) con copia al correo de su país: [vascularstudycolombia@gmail.com](mailto:vascularstudycolombia@gmail.com) / [estudiovascularargentina@gmail.com](mailto:estudiovascularargentina@gmail.com) / vascularstudymexico@gmail.com/ [vascularsudychile@gmail.com](mailto:vascularsudychile@gmail.com) ).

**Muchas Gracias!**
