# Supplementary material for: Peripheral vascular catheter use in Latin America (the vascular study): A multinational cross-sectional study
Source: Front Med (Lausanne). 2023 Jan 4;9:1039232. doi: 10.3389/fmed.2022.1039232 (PMC9846050; doi:10.3389/fmed.2022.1039232)
Supplement: Supplementary file 1 [file Data_Sheet_1.zip › Supplementary File 5.DOCX]

**SUPPLEMENTARY FILE 5 – DATA COLLECTION FORM**

Form 4 – Data collection form in English version.

| **Hospital/Site** |  | |
| --- | --- | --- |
| **Ward/Unit** |  | |
| **Health speciality** | - **Medical** - **Surgical** - **Oncology/Haematology** - **Intensive Care Unit** - **Emergency Department Coronary Care Unit** - **High-Dependency / Step-Down Unit** - **Obstetrics** - **Day Stay / Short Stay Unit** - **Other ____________________** | |
| **Room/Bed number** |  | |
| **Screening log number** |  | |
| **Age of patient** |  | |
| **Gender of patient** | 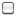 Male | 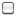 Female |
| **Date and time of review** | / / 2018 ____:____ am/pm | |

**VASCULAR PIVC Study**

**Data Collection Form**

*Please obtain verbal consent and complete a separate survey for each PIVC. Thank-you!*

PIVC: short peripheral intravenous catheter

This form should contain no identifying patient information.

| **Date of PIVC insertion (Ask patient if not documented)** | | | | | | **Time of insertion (Ask patient if not documented)** | | | | |
| --- | --- | --- | --- | --- | --- | --- | --- | --- | --- | --- |
| _______/_______/ 2018 | | | 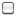 Not documented | | | _____:_____ am/pm | | | 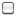 Not documented | |
| day month | | | | | |  | |  | | |
| **Reason for PIVC insertion (check all that apply)** | | | | | | | | | | |
| 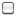 | | IV fluids | | | | 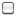 | | Blood product transfusion | | |
| 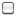 | | IV medications | | | | 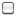 | | Parenteral nutrition | | |
| 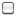 | | Taking blood | | | | 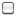 | | Chemotherapy | | |
| 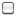 | | Patient unstable / Requiring resuscitation | | | | 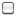 | | Unknown | | |
| **Who inserted the catheter? (Ask patient if not documented)** | | | | | | **Where was catheter inserted? (Ask patient if not documented)** | | | | |
| 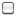 | | IV team | | | | 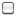 | | Emergency department | | |
| 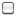 | | Registered Nurse | | | | 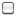 | | Operating room | | |
| 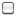 | | Nurse Technician | | | | 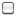 | | Ward | | |
| 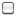 | | Auxiliary Nurse | | | | 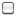 | | ICU/CCU | | |
| 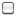 | | Paramedic | | | | 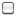 | | Clinic | | |
| 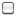 | | Doctor | | | | 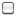 | | Radiology/Procedure room | | |
| 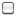 | | Student | | | | 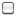 | | Off-site (e.g., ambulance, other hospital etc) | | |
|  | |  | | | | 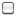 | | Unknown/Not documented | | |
| 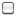 | | Other _________________________ | | | |  | |  | | |
| 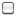 | | Unknown/Not documented | | | |  | |  | | |
| **Catheter type (refer to Product Guide)** | | | | | | **How many insertion attempts were required (Ask patient if not documented in the chart)** | | | | |
| 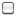 | Winged | | | | | 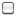 | | One | | |
| 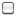 | Ported | | | | | 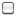 | | Two | | |
| 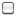 | Winged with integrated extension | | | | | 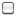 | | Three or more | | |
| 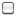 | Non-winged/non-ported | | | | | 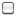 | | Unknown | | |
| **PIVC position/site** | | | | | | **Catheter gauge/size** | | | | |
| 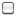 | | Hand | | 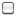 | Upper arm | 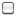 | | 14 G (orange) | 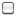 | 22 G (blue) |
| 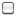 | | Wrist | | 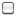 | Foot | 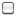 | | 16 G (grey) | 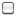 | 24 G (yellow) |
| 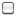 | | Forearm | | 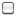 | Head | 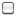 | | 18 G (green) | 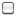 | 26 G (purple) |
| 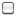 | | Antecubital fossa | |  |  | 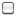 | | 20 G (pink) | 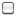 | Not visible |
| 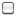 | | Other _________________________ | | | | 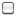 | | Other ___________________________ | | |
| **PIVC site assessment (check all that apply)**  * Advise patient’s nurse of these findings | | | | | | | | | | |
| 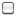 | | No clinical symptoms | | | | 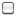 | | Palpable hard vein cord beyond IV tip * | | |
| 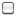 | | Pain/ * | | | | 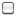 | | Streak/red line along vein * | | |
| 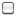 | | Tenderness on palpation* | | | | 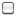 | | New Fever | | |
| 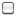 | | Redness > 1 cm from insertion site* | | | | 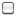 | | Induration/hardness of tissues > 1 cm * | | |
| 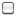 | | Swelling > 1 cm from insertion site * | | | | 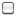 | | Leaking PIVC * | | |
| 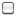 | | Purulence * | | | | 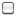 | | Extravasation/infiltration * | | |
| 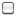 | | Itch / rash under dressing * | | | | 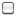 | | Blood in line | | |
| 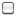 | | Blistering/skin tears under dressing * | | | | 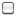 | | Partial/complete dislodgement of PIVC * | | |
| 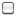 | | Bruising | | | | 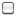 | Other ___________________________ | | | |
| 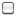 | | Dried blood around PIVC | | | |  |  | | | |
| Has a PIVC site assessment been documented in the patient chart in last 24 hours? 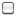 Yes 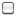 No | | | | | | | | | | |
| Has the PIVC been used in the last 24 hours? 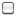 Yes 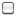 No 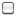 Yes 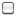 No | | | | | | | | | | |
| Is it likely that the PIVC will be used in the next 24 hours? 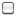Yes 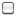 No  (e.g. upcoming procedure, telemetry, unstable patient, etc) | | | | | | | | | | |

| **Ward/Unit _____________________** | | **Room/Bed number _____________________** | |
| --- | --- | --- | --- |
| **IV dressing type (refer to dressing guide)** | | **IV dressing assessment** | |
| 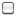 | Borderless transparent polyurethane dressing ^1^ | 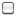 | Clean, dry and intact |
| 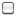 | Window transparent polyurethane dressing ^2^ | 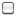 | Moist and soiled with blood/discharge |
| 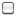 | Sterile gauze and tape dressing ^3^ | 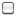 | Dry and soiled with blood/discharge |
| 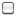 |  | 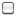 | Loose or lifting edges |
| 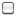 | Tape only | 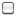 | Other ____________________________ |
| 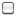 | Other __________________________ |  |  |
| 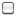 | No dressing |  |  |

| **PIVC & administration set securement**  **(check all that apply) (refer to Product Guide)** | | **IV connectors and tubing (check all that apply) (refer to Product guide)** | | |
| --- | --- | --- | --- | --- |
| 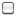 | Sutureless securement device ^5^ | 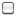 | | Extension tubing ^7^ |
| 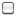 | Sterile tape strips around PIVC | 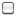 | | Stopcock/3-way tap ^8^ |
| 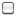 | Non-sterile tape around PIVC | 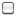 | | Needleless connector/IV bung ^9^ |
| 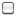 | Non-sterile tape over PIVC dressing |  | | IV end cap ^10^ |
|  | Non-sterile tape around administration set |  | | IV administration set |
|  | IV administration set securement device ^6^ |  | | Other __________________________ |
|  | Splint/bandage/tubular net |  | | None |
|  | Site dressing only |  | |  |
|  | Other ___________________________ |  | |  |
|  | No securement |  | |  |
| **IV fluids today (check all that apply)** | | | **Rate of Infusion** | |
|  | Crystalloid (e.g. normal saline, 5% dextrose) |  | | Continuous infusion |
|  | Colloid or blood products |  | | Intermittent infusion |
|  | Parenteral nutrition |  | | Bolus injection |
|  | None |  | | Combination of intermittent and bolus |
|  | Not documented / chart unavailable |  | |  |
| **If the patient receives an IV flush bolus to keep PIVC patent, what is the flush solution used?** | | | **If the patient receives an IV flush bolus to keep PIVC patent, how often is the PIVC flushed?** | |
|  | 0.9% sodium chloride |  | | NA – Continuous infusion |
|  | Heparin/heparinized saline solution |  | | Every 4 hours |
|  | __________ units/mL |  | | Every 8 hours |
|  | Other |  | | Every 12 hours |
|  | ____________________________ |  | | Once daily |
|  | No order |  | | After medication |
|  |  |  | | Not documented |
|  |  |  | | Other ____________________________ |
| **IV medications today (via PIVC) (check all that apply)** | | | | |
|  | Electrolytes (KCl, Mg, etc.) |  | | Heparin infusion |
|  | Antibiotics *List*____________________________ |  | | Insulin |
|  | Analgesia/PCA |  | | Gastric protection |
|  | Sedation |  | | Anti-convulsant |
|  | Diuretic |  | | Chemotherapy |
|  | Anti-emetic |  | | Other ___________________________None |
| **Please ask the patient the following question:**  What has been your experience with this IV catheter? 0 = Worst possible 10 = Best possible ___________  Patient cannot verbalise/understand | | | | |
| Please enter your completed data on the on-line survey link provided via email, or contact us for postage details, or scan and email completed form to m.arneil@griffith.edu.au | | | | |

Form 5 – Data collection form in Portuguese version.

| **Estudo VASCULAR Formulário de Coleta de Dados**  *Por favor, complete a pesquisa separada para cada CIP*.*  *Obrigado!*  **CIP: cateter intravenoso periférico.*  Esse formulário não deve conter nenhuma informação que identifique o paciente. | | | **Hospital** | | |  | | |
| --- | --- | --- | --- | --- | --- | --- | --- | --- |
|  |  |  | **Nome da Setor ou**  **Unidade** | | |  | | |
|  |  |  | **Especialidades Médicas** | | | - **Clínica** - **Cirúrgica** - **Oncologia/Hematologia** - **Cuidado Intensivo** - **Departamento de emergência** - **Unidade Coronariana** - **Unidade Semi-Intensiva** - **Obstetrícia/Ginecologia** - **Hospital dia/Unidade de curta permanência** - **Outros _** | | |
|  |  |  | **Quarto/ Leito** | | |  | | |
|  |  |  | **Nº do paciente no**  **”Formulário de Triagem**  **do Serviço/Unidade”** | | |  | | |
|  |  |  | **Data de Nascimento do**  **paciente** | | |  | | |
|  |  |  | **Sexo do paciente** | | | - Masculino - Outro | | - Feminino |
|  |  |  | **Data e hora da coleta** | | | / /20 : am/pm | | |
| **Data de inserção do CIP** | | | | **Hora de inserção do CIP** | | | | |
| _/ / 20 | | Não documentado | | :_ am/pm | | | Não documentado | |
| dia mês | |  | |  |  |  |  | |
| **Motivo para inserção do CIP (assinale as opções aplicáveis)** | | | | | | | | |
|  | Fluidos IV |  |  |  | Transfusão de produtos sanguíneos | | | |
|  | Medicação IV |  |  |  | Nutrição parenteral | |  |  |
|  | Coleta de sangue |  |  |  | Quimioterapia | |  |  |
|  | Paciente instável / Condição de emergência | | |  | Outro _ | | | |
|  |  | | |  | Desconhecido | |  |  |
| **Quem inseriu o CIP?** | | | | **Local de realização do procedimento** | | | | |
|  | Equipe de Terapia Intravenosa | |  |  | Setor de emergência | | |  |
|  | Enfermeiro |  |  |  | Centro Cirúrgico/ Centro Obstétrico | | | |
|  | Técnico de enfermagem | |  |  | Enfermaria/ Quartos | | |  |
|  | Auxiliar de enfermagem | |  |  | Unidade de Cuidados Intensivos / Unidade  Coronariana | | | |
|  | Médico |  |  |  | Ambulatório (Clinic) | | |  |
|  | Estudante de Enfermagem | |  |  | Radiologia/ Sala de procedimentos | | | |
|  | Estudante de Medicina | |  |  | Fora da instituição (ex.: ambulância, outro hospital, etc.) | | | |
|  | Desconhecido/Não documentado | |  |  | Desconhecido | |  |  |
|  | Outro _ | | |  |  | |  |  |
| **Tipo do cateter (consultar guia de produtos)** | | | | **Quantas tentativas para inserção do CIP foram**  **necessárias?** | | | | |
|  | Com asas^1^ |  |  |  | Uma (primeira tentativa com sucesso) | | | |
|  | Com portal^2^ |  |  |  | Duas (segunda tentativa com sucesso) | | | |
|  | Agulhado com asas e extensão^3^ | |  |  | Três ou mais | |  |  |
|  | Sobre agulha com asas e extensão^4^ | |  |  |  | |  |  |

|  | Sem asa/sem portal^5^ |  |  |  | Desconhecido |  |  |
| --- | --- | --- | --- | --- | --- | --- | --- |
|  | Outro _ | | |  |  |  |  |
| **Local de inserção/ Posição do CIP** | | | | **Tamanho do cateter** | | | |
|  | Mão |  | Braço |  | 14 G (laranja) |  | 22 G (azul) |
|  | Punho |  | Pé |  | 16 G (cinza) |  | 24 G (amarelo) |
|  | Antebraço |  | Perna |  | 18 G (verde) |  | 26 G (roxo) |
|  | Fossa antecubital |  | Cabeça |  | 20 G (rosa) |  | Não visível |
|  | Outro _ | | |  | Outro _ | | |
| **Observação do local do CIP (assinalar as opções aplicáveis coletando as informações no prontuário, com a**  **equipe de saúde e/ou observação)** *Caso você identifique qualquer alteração durante a observação, informar a equipe de saúde. | | | | | | | |
|  | Sem sinais clínicos |  |  |  | Sangue seco ao redor do CIP | |  |
|  | Dor* |  |  |  | Cordão venoso palpável* | |  |
|  | Doloroso à palpação* | |  |  | Trajeto venoso hiperemiado* | | |
|  | Hiperemia > 1 cm do local de inserção* | | |  | Endurecimento do tecido > 1 cm* | | |
|  | Edema > 1 cm do local de inserção* | | |  | Vazamento ao redor da inserção do CIP* | | |
|  | Presença de pus* |  |  |  | Extravasamento/infiltração* | |  |
|  | Hiperemia, rash/erupção sob o curativo* | | |  | Sangue na extensão do sistema de infusão | | |
|  | Bolhas/vesículas sob o curativo* | | |  | Deslocamento parcial ou completo do CIP* | | |
|  | Hematoma |  |  |  | Outros _ | | |
| Houve registro da avaliação do sítio de inserção do cateter nas últimas 24h? Sim Não | | | | | | | |
| O CIP foi utilizado nas últimas 24 horas? Sim Não | | | | | | | |
| É provável que se use o CIP nas próximas 24 horas? Sim Não (ex.: procedimento próximo, cirurgias ou exames, etc.) | | | | | | | |

| **Tipo de curativo/cobertura do CIP (consultar guia de**  **produtos)** | | **Observações do curativo IV** | |
| --- | --- | --- | --- |
|  | Película transparente simples^6^ |  | Limpo, seco e intacto |
|  | Película transparente em poliuretano com borda^7^ |  | Com sangue ou outros fluidos/secreção |
|  | Gaze estéril com fita adesiva^8^ |  | Seco e sujo com sangue |
|  | Curativo impregnado com clorexidina^9^ |  | Solto ou bordas dobradas |
|  | Fita adesiva apenas |  | Outros |
|  | Outros |  |  |
|  | Sem curativo |  |  |
| **Estabilização/Fixação CIP e do Sistema de infusão**  **(assinale as opções aplicáveis) (ver guia)** | | **Extensões e conexões IV (assinale as opções**  **aplicáveis) (consultar guia de produtos)** | |
|  | Dispositivo de fixação sem sutura^10^ |  | Tubos extensores^12^ |
|  | Fitas adesivas estéreis em torno do CIP |  | Torneirinhas de 3 vias^13^ |
|  | Fita adesiva não estéril em torno do CIP |  | Conectores sem agulha/valvulado^14^ |
|  | Fita adesiva não estéril por cima da cobertura do CIP |  | Tampinha^15^ |
|  | Fita adesiva não estéril em torno sistema de  infusão/extensões |  | Conexão direta ao sistema de infusão |
|  | Dispositivo de fixação do sistema de infusão^11^ |  | Nenhum |
|  | Tala/Bandagem/Malha tubular |  | Outro |
|  | Sem fixação do sistema de infusão |  |  |
|  | Outro _ |  |  |

| **Soluções IV administradas nas últimas 24 horas (assinalar**  **as opções aplicáveis)** | | **Tipo de Infusão IV** | |
| --- | --- | --- | --- |
|  | Cristalóide (ex.: solução salina, glicose) |  | Infusão contínua |
|  | Colóide ou produtos sanguíneos |  | Infusão intermitente |
|  | Nutrição parenteral |  | Infusão em *bolus* |
|  | Nenhuma |  | Combinação intermitente e *bolus* |
|  | Não documentado / Prescrição indisponível |  | Apenas *flush* |
|  |  |  | Outro _ |
| **Solução utilizada para manutenção intermitente (permeabilização) do CIP?** | | **Caso faça a manutenção intermitente, qual a frequência?** | |
|  | Soro Fisiológico |  | NA – Infusão contínua |
|  | Heparina/solução heparinizada |  | A cada 4 horas |
|  | Outro _ |  | A cada 8 horas |
|  | Nenhum |  | A cada 12 horas |
|  |  |  | Uma vez ao dia |
|  |  |  | Após as medicações |
|  |  |  | Não documentado |
|  |  |  | Outro _ |
| **Medicamentos IV administrados nas últimas 24 horas (via CIP) (assinale as opções aplicáveis)** | | | |
|  | Eletrólitos (KCl, Mg, etc.) |  | Infusão com heparina |
|  | Antibiótico  Listar: |  | Insulina |
|  | Analgésico/PCA |  | Protetor gástrico |
|  | Sedativo |  | Anticonvulsivante |
|  | Diurético |  | Quimioterápico |
|  | Antiinflamatório |  | Nenhum |
|  | Antiemético |  | Outro |

Por favor, introduza os dados completos no link da pesquisa on-line fornecido via e-mail ou, se desejar mais informações sobre a postagem, escaneie e envie por e-mail o formulário preenchido para [vascularstudybrazil@gmail.com.](mailto:vascularstudybrazil@gmail.com)

Contate-nos para qualquer detalhe.

Obrigado!

Form 6 – Data collection form in Spanish version.

Estudio VASCULAR

**Formulario de Recolección de Datos**

*Por favor complete una encuesta separada para cada catéter venoso periférico corto CVPC*. Gracias!*

*CVPC (PIVC): catéter venoso periférico corto

Este formulario no debe contener información que identifique al paciente.

***número del paciente en el formulario de “Registro de pacientes**

| **Hospital/Sitio** |  | |
| --- | --- | --- |
| **Nombre del Servicio**  **o Unidad** |  | |
| **Especialidad médica** | - **Clínica Médica/Hospitalización** - **Quirúrgico/Cirugía** - **Oncología/Hematología** - **Cuidado Intensivo** - **Departamento de urgencias** - **Unidad de cuidado coronario** - **Cuidado Intermedio** - **Obstetricia/Ginecología** - **Hospital día** - **Otros** | |
| **Habitación/Nro. De**  **cama/suite** |  | |
| **Número de registro*** |  | |
| **Fecha de nacimiento**  **del paciente** |  | |
| **Género del paciente** | - Masculino - Otro | - Femenino |
| **Fecha y hora de la**  **evaluación** | / / 20 : | |

**del servicio/unidad seleccionada”**

| **Fecha de inserción del CVPC**  **(preguntar al paciente si no está registrado)** | | | | | **Hora de inserción del CVPC**  **(preguntar al paciente si no está registrado)** | | | | | |  |  |
| --- | --- | --- | --- | --- | --- | --- | --- | --- | --- | --- | --- | --- |
| /_ / 20 | | | No documentado | | :_ | | | No documentada | | |  |  |
| Día Mes | | |  | |  |  |  |  | | |  |  |
| **Razones para la inserción del CVPC (marcar todo lo que corresponde)** | | | | | | | | | | |  |  |
|  | Fluidos IV | |  | |  | | Transfusión de productos sanguíneos | | | |  |  |
|  | Medicación IV | |  | |  | | Nutrición parenteral | | | |  |  |
|  | Extracción de sangre | |  | |  | | Quimioterapia |  | | |  |  |
|  | Paciente inestable / Requiere resucitación | | | |  | | Otros _ | | | |  |  |
|  |  | | | |  | | No se sabe |  | | |  |  |
| **¿Quién ha colocado el CVPC?**  **(preguntar al paciente si no está registrado)** | | | | | **¿En qué servicio se insertó el catéter?**  **(preguntar al paciente si no está registrado)** | | | | | |  |  |
|  | Equipo IV | |  | |  | | Departamento de Emergencias | | | |  |  |
|  | Enfermero/ Licenciado en Enfermero | | | |  | | Quirófano / Sala de operaciones/ Centro Obstétrico | | | |  |  |
|  | Técnico (diagnóstico/hemoterapia/anestesia) | | | |  | | UCI/UCC |  | | |  |  |
|  | Auxiliar de enfermería | | | |  | | Clínica Médica/ hospitalization | | | |  |  |
|  | Paramédico | |  | |  | | Radiología/Sala de procedimientos | | | |  |  |
|  | Médico/Residente | |  | |  | | Fuera del lugar (ej., ambulancia, otro hospital etc.) | | | |  |  |
|  | Estudiante de Enfermería | | | |  | | No se sabe |  | | |  |  |
|  | Estudiante de Medicina | | | |  | |  |  | | |  |  |
|  | Otro _ | | | |  | |  |  | | |  |  |
|  | No se sabe | |  | |  | |  |  | | |  |  |
| **Tipos de Catéter (consulte la Guía de Productos)** | | | | | **¿Cuántos intentos de inserción fueron requeridos?**  **(preguntar al paciente si no está registrado)** | | | | | |  |  |
|  | Con alas^1^ | |  | |  | | Uno (primer intento con éxito) | | | |  |  |
|  | Con portal^2^ | |  | |  | | Dos (Segundo intento con éxito) | | | |  |  |
|  | Con aguja, alas y extensión^3^ | | | |  | | Tres o más |  | | |  |  |
|  | Sobre la aguja, con alas y sistema cerrado integrado^4^ | | | |  | | No se sabe |  | | |  |  |
|  | Sin alas/sin portal^5^ |  | |  |  | | | | | |  |  |
|  | Otro _ | | | |  |  |  |  |  |  |  |  |
| **Ubicación (posición/sitio) del CVPC** | | | | | **Catéter calibre/tamaño** | | | | | |  |  |
|  | Mano |  | | Brazo |  | | 14 G (naranja) | |  | 22 G (azul) |  |  |
|  | Muñeca |  | | Pie |  | | 16 G (gris) | |  | 24 G (amarillo) |  |  |
|  | Antebrazo |  | | Pierna |  | | 18 G (verde) | |  | 26 G (púrpura) |  |  |
|  | Fosa antecubital |  | | Cabeza |  | | 20 G (rosa) | |  | No visible |  |  |
|  | Otro _ | | | |  | | Otro _ | | | |  |  |
| **Evaluación del sitio del CVPC (marcar todo lo que corresponda)** *Avisar al enfermero del paciente de estos hallazgos | | | | | | | | | | |  |  |
|  | Sin síntomas clínicos |  | |  |  | | Sangre seca alrededor del CVPC | | | |  |  |
|  | Dolor* |  | |  |  | | Cordón venoso indurado palpable más allá del  extremo/punta del CVPC* | | | |  |  |
|  | Sensibilidad a la palpación* | | |  |  | | Línea roja a lo largo de la vena* | | | |  |  |
|  | Enrojecimiento > 1 cm del sitio de inserción* | | | |  | | Induración / tejidos endurecido > 1 cm* | | | |  |  |
|  | Inflamación > 1 cm del sitio de inserción* | | | |  | | Pérdida/fuga alrededor de la inserción* | | | |  |  |
|  | Purulencia* |  | |  |  | | Extravasación/infiltración * | | |  |  |  |
|  | Prurito/rash/erupción debajo de la curación* | | | |  | | Sangre en la tubuladura/equipo de infusión | | | |  |  |
|  | Ampollas/piel lesionada debajo de la curación* | | | |  | | Desplazamiento parcial/completo del CVPC* | | | |  |  |
|  | Hematoma |  | |  |  | | Otros | | | |  |  |
| ¿Se ha documentado evaluación del sitio CVPC en las últimas 24 horas? Sí No | | | | | | | | | | |  |  |
| ¿Se ha usado el CVPC en las últimas 24 horas? Sí No | | | | | | | | | | |  |  |
| Es probable que el CVPC se use en las próximas 24 horas? Sí No (ejemplo: próximo procedimiento, cirugías, examenes, etc.) | | | | | | | | | | |  |  |
| **Tipo de Curación/Vendaje IV (consulte la guía de vendaje)** | | | | | | | **Evaluaciónes de la Curación/vendaje IV** | | | | | |
| Apósito de poliuretano transparente sencillos ^6^ Apósito de poliuretano transparente con bordes ^7^ Gasa estéril y vendaje de cinta^8^  Apósito impregnado con clorhexidina^9^  Tela adhesiva solamente  Otros Ninguno | | | | | | | Limpio, seco, intacto  Húmedo y sucio/manchado con sangre/otro fluido- secreción  Seco y sucio/manchado con sangre/otro fluido- secreción  Bordes sueltos o levantados  Otros _ | | | | | |
| **Aseguramiento (marque todo lo que corresponda) (consulte la guía del producto)** | | | | | | | **Conectores intravenosos en uso**  **(marcar todo lo que corresponda) (consulte la guía del**  **producto)** | | | | | |
| Dispositivo de sujeción sin suturas ^10^ | | | | | | | Tubuladura/equipo de extensión/prolongador ^12^ | | | | | |
| Cintas estériles alrededor del CVPC | | | | | | | Llave de 3 vías ^13^ | | | | | |
| Cinta adhesiva NO estéril alrededor del CVPC | | | | | | | Conector sin aguja ^14^ | | | | | |
| Cinta adhesiva NO estéril sobre la curación/ apósito  del CVPC  Cinta adhesiva NO estéril alrededor del set de  administración/tubuladura/equipo | | | | | | | Tapón/Tapa IV ^15^  Conexión directa con la tubuladura/equipo/set de administración | | | | | |
| Dispositivo de sujeción de tubuladura/equipo IV^11^ | | | | | | | Otros _ | | | | | |
| Tablilla/vendaje/malla-red tubular | | | | | | | Ninguno | | | | | |
| Sin aseguramiento | | | | | | |  | | | | | |
| Otros | | | | | | |  | | | | | |

| **Fluidos IV de las ultimas 24 horas (marcar todo lo que corresponda)** | | **Tipo de Infusión (marcar todo lo que corresponda)** | |
| --- | --- | --- | --- |
|  | Cristaloides (ej.: solución salina normal, dextrosa al  5%) |  | Infusión continua |
|  | Productos sanguíneos |  | Infusión intermitente |
|  | Nutrición parenteral |  | Injección en bolos |
|  | Ninguno |  | Combinación intermitente y bolos |
|  | No documentada/registros no disponibles |  | *Flush* solamente |
|  |  |  | Otros |
| **Si el paciente tiene lavados para mantener la permeabilidad de la vía, ¿Cuál es la solución de lavado?** | | **Si el paciente tiene lavados para mantener la permeabilidad de la vía, ¿Cuál es la frecuencia de lavados**  **de la cánula/catéter?** | |
|  | Cloruro de sodio 0,9% |  | NA – Infusión continua |
|  | Solución salina heparinizada |  | Cada 4 horas |
|  | Otros _ |  | Cada 8 horas |
|  | Ninguno |  | Cada 12 horas |
|  |  |  | Una vez al día |
|  |  |  | Después de medicación |
|  |  |  | No documentada |
|  |  |  | Otros _ |
| **Medicación IV de las últimas 24 horas (vía CVPC) (marcar todo lo que corresponda)** | | | |
|  | Electrolitos (KCl, Mg, etc.) |  | Infusión de heparina |
|  | Listar los Antibioticos:  _ |  | Insulina |
|  | Analgesia / Analgesia controlada por el paciente |  | Protección gástrica |
|  | Sedación |  | Anti-convulsivante |
|  | Diurético |  | Quimioterapia |
|  | Anti-inflamatorio |  | Otros |
|  | Antiemético |  | Ninguno |
| **Por favor preguntar al paciente:**  ¿Cuál ha sido su experiencia con el catéter IV? 0 = la peor posible 10 = la mejor posible _ El paciente no puede entender/hablar | | | |

Por favor introduzca los datos completos en la página web de la encuesta por medio del vínculo enviado por mail, o contáctenos para los detalles de envío postal, o escaneo y envío por correo electrónico a [vascularstudybrazil@gmail.com](mailto:vascularstudybrazil@gmail.com) con copia al correo a [vascularstudyargentina@gmail.com](mailto:vascularstudyargentina@gmail.com), [vascularstudychile@gmail.com](mailto:vascularstudychile@gmail.com) o [vascularstudymexico@gmail.com.](mailto:vascularstudycolombia@gmail.com)

Muchas Gracias!
