# Supplementary material for: Peripheral vascular catheter use in Latin America (the vascular study): A multinational cross-sectional study
Source: Front Med (Lausanne). 2023 Jan 4;9:1039232. doi: 10.3389/fmed.2022.1039232 (PMC9846050; doi:10.3389/fmed.2022.1039232)
Supplement: Supplementary file 1 [file Data_Sheet_1.zip › Supplementary File 6.DOCX]

**SUPPLEMENTARY FILE 6 – REASEARCH QUESTIONS AND DATA COLLLECTION AS PER VHP MODEL**

| **Hospital Policy – Site Information Survey** | **PIVC Audit – Data Collection** |
| --- | --- |
| **Study questions** | |
| 1. What are the hospital policies and processes for PIVC insertion and maintenance? | 1. What are the PIVC inserter characteristics (level of education, training)? 2. What is the prevalence of PIVC complications (pain, redness, and edema)? 3. What is the prevalence of redundant/idle PIVCs (calculated by number of PIVCs not used for intravenous therapy in a 24-hour period)? |
| **VHP Model components and data collection** | |
| **4. Evaluation** | **1. Assessment/selection** |
| Frequency of PIVC replacement in adults:   - every 8–72 hours - every 72–96 hours - clinically indicated   Frequency of PIVC replacement in children:   - every 8–72 hours - every 72–96 hours - clinically indicated   Frequency of site assessment:   - every 6 hours - every 8 hours - every 12 hours - every 24 hours - whenever the catheter is used   Recommended PIVC insertion technique:   - clean - aseptic (non-touch technique) - strictly aseptic (using sterile glove)   Clinicians who insert PIVCs:   - specialist IV team - nurse - nursing technician - nursing assistant - nursing student - doctor/resident - medical student   Recommended PIVC dressing type:   - borderless transparent sterile polyurethane - window transparent sterile polyurethane - sterile gauze and tape - sterile tape - non-sterile tape - chlorhexidine-impregnated sterile sponge/polyurethane - others   Cleaning solution used for insertion (including concentrations):   - chlorhexidine in alcohol - povidone-iodine - alcohol - 0.9% sodium chloride | Reason for insertion:   - intravenous medications - intravenous fluids   Catheter type:   - non-winged/non-ported - ported - plastic-winged needle with extension   Catheter size:   - large ≥18 gauge - medium 20–22 gauge - small ≤24 gauge |
|  | **2. Insertion** |
|  | Inserter:   - nurse - nursing assistant - nurse technician - doctor   Insertion location:   - medical/surgical ward - emergency department - intensive/critical care unit   Number of attempts:   - one - two - three or more - unknown   Dressing applied:   - simple transparent polyurethane sterile dressing - bordered transparent polyurethane sterile dressing - non-sterile tape only |
|  | **3. Management** |
|  | PIVC site assessment documented in previous 24 hours?  PIVC not used (redundant) in previous 24 hours?  Assessment of dressing:   - clean/dry/intact - moist or soiled with blood - soiled/stained with blood   Assessment of PIVC site:   - blood in tubing - pain - pain on palpation - dried blood around PIVC - redness (hyperemia) >1cm - edema >1cm |
